# Identification of residues important for the activity of aldehyde-deformylating oxygenase through investigation into the structure-activity relationship

**DOI:** 10.1186/s12896-017-0351-8

**Published:** 2017-03-16

**Authors:** Qing Wang, Luyao Bao, Chenjun Jia, Mei Li, Jian-Jun Li, Xuefeng Lu

**Affiliations:** 1 Key Laboratory of Biofuels, Shandong Provincial Key Laboratory of Energy Genetics, Qingdao Institute of Bioenergy and Bioprocess Technology, Chinese Academy of Sciences, Qingdao, 266101 China; 2 University of Chinese Academy of Sciences, Beijing, 100049 China; 3 National Laboratory of Biomacromolecules, Institute of Biophysics, Chinese Academy of Sciences, Beijing, 100101 China; 4 Present Address: National Key Laboratory of Biochemical Engineering, Institute of Process Engineering, Chinese Academy of Sciences, Beijing, 100190 China

**Keywords:** Aldehyde-deformylating oxygenase, Site-directed mutagenesis, Structure-activity relationship, Fatty alk(a/e)ne, *Synechococcus elongatus* PCC7942, *Synechocystis* sp. PCC6803

## Abstract

**Background:**

Aldehyde-deformylating oxygenase (ADO) is a key enzyme involved in the biosynthetic pathway of fatty alk(a/e)nes in cyanobacteria. However, cADO (cyanobacterial ADO) showed extreme low activity with the *k*_*ca*t_ value below 1 min^−1^, which would limit its application in biofuel production. To identify the activity related key residues of cADO is urgently required.

**Results:**

The amino acid residues which might affect cADO activity were identified based on the crystal structures and sequence alignment of cADOs, including the residues close to the di-iron center (Tyr39, Arg62, Gln110, Tyr122, Asp143 of cADO-1593), the protein surface (Trp 178 of cADO-1593), and those involved in two important hydrogen bonds (Gln49, Asn123 of cADO-1593, and Asp49, Asn123 of cADO-sll0208) and in the oligopeptide whose conformation changed in the absence of the di-iron center (Leu146, Asn149, Phe150 of cADO-1593, and Thr146, Leu148, Tyr150 of cADO-sll0208). The variants of cADO-1593 from *Synechococcus elongatus* PCC7942 and cADO-sll0208 from *Synechocystis* sp. PCC6803 were constructed, overexpressed, purified and kinetically characterized. The *k*_*cat*_ values of L146T, Q49H/N123H/F150Y and W178R of cADO-1593 and L148R of cADO-sll0208 were increased by more than two-fold, whereas that of R62A dropped by 91.1%. N123H, Y39F and D143A of cADO-1593, and Y150F of cADO-sll0208 reduced activities by ≤ 20%.

**Conclusions:**

Some important amino acids, which exerted some effects on cADO activity, were identified. Several enzyme variants exhibited greatly reduced activity, while the *k*_*cat*_ values of several mutants are more than two-fold higher than the wild type. This study presents the report on the relationship between amino acid residues and enzyme activity of cADOs, and the information will provide a guide for enhancement of cADO activity through protein engineering.

**Electronic supplementary material:**

The online version of this article (doi:10.1186/s12896-017-0351-8) contains supplementary material, which is available to authorized users.

## Background

Fatty alk(a/e)nes, which can be produced by plants, insects, birds, green algae, and cyanobacteria, are the main components of conventional fuels, and have been considered as the ideal replacement for fossil-based fuels [1–5]. It has been accepted that a two-step pathway for fatty alk(a/e)ne biosynthesis exists, involving reduction of fatty acyl-ACP or -CoA into corresponding aldehyde by acyl-ACP reductase and conversion of fatty aldehyde into alk(a/e)ne by aldehyde decarbonylase. In 2010, Schirmer et al. identified two genes involved in fatty alk(a/e)ne biosynthesis in cyanobacteria: acyl-ACP reductase and aldehyde decarbonylase (renamed as aldehyde-deformylating oxygenase, ADO) [1, 6]. Since then cADO (cyanobacterial ADO) has attracted particular interest due to the difficult and unusual reaction it catalyses [7].

**Fig. 1:**
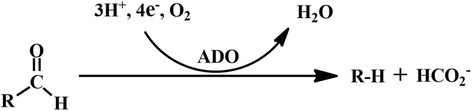
cADO-catalyzed reaction [12–16]

The crystal structures of cADO revealed that cADO belongs to the non-heme dinuclear iron oxygenase family of enzymes exemplified by methane monoxygenase, type I ribonucleotide reductase, and ferritin. The di-iron center is contained within an antiparallel four-α-helix bundle, where two histidines and four carboxylates (aspartate or glutamate) supply the protein ligands to the metal ions [1, 8–11]. The C1-derived co-product of the cADO-catalyzed reaction is formate (Fig. 1) [12]. Oxygen is needed, and one O-atom is incorporated into formate [13]. The auxiliary reducing system (biological or chemical) providing four electrons is required, and the endogenous electron transfer system worked more effectively than the heterologous and chemical ones in supporting cADO activity [1, 12, 14–16]. Self-sufficient cADOs fused to homogenous ferredoxin and ferredoxin-NADP^+^ reductase could efficiently catalyze the conversion of aldehydes into alk(a/e)nes [17]. It has been found that cADO also produces *n*-1 aldehydes and alcohols in addition to alk(a/e)ne [18]. Mechanistic studies have demonstrated that a radical intermediate is involved in the cADO-catalyzed reaction, and a possible catalytic process has been proposed based on the crystal structures of cADO from *Synechococcus elongates* strain PCC7942 [9, 19–21]. Moreover, cADO was engineered to improve specificity for short- to medium-chain aldehydes [22]. Recently, Hayashi et al. investigated the role of three cysteine residues of cADO in the structure, stability and alk(a/e)ne production [23].

However, cADO showed extreme low activity with the *k*_*ca*t_ value below 1 min^−1^, which would present a major barrier to its application in biofuel production [8, 14–17, 22]. In order to address this issue, protein engineering of cADO for improved activity including rational design and/or directed evolution is urgently needed. The knowledge about structure-function relationship of cADOs is the prerequisite for rational protein engineering. Until now, no detailed studies towards structure-function relationship of cADOs have been carried out. Some crystal structures of cADOs from *P. marinus* strain MIT9313 and *Synechococcus elongates* strain PCC7942 have been resolved, which have provided a base for identification of the residues important for cADO activity [8, 9].

In the current study, some amino acids which might affect cADO activity were identified through analysis of the cADO crystal structures and sequence alignment of some cADOs. The corresponding enzyme variants were made and characterized. We have found some essential residues for the cADO-catalyzed reaction, which will lay the foundation for improvement of cADO activity through protein engineering.

## Results

### Identification of the target residues for mutagenesis

The following residues were identified and investigated in the current study. Since the variants including mutations of the residues involved in coordinating the di-iron center negatively affected cADO activity, they were not included in the current study [9].

#### Residues whose conformations have changed in the absence of the di-iron center

**Fig. 2:**
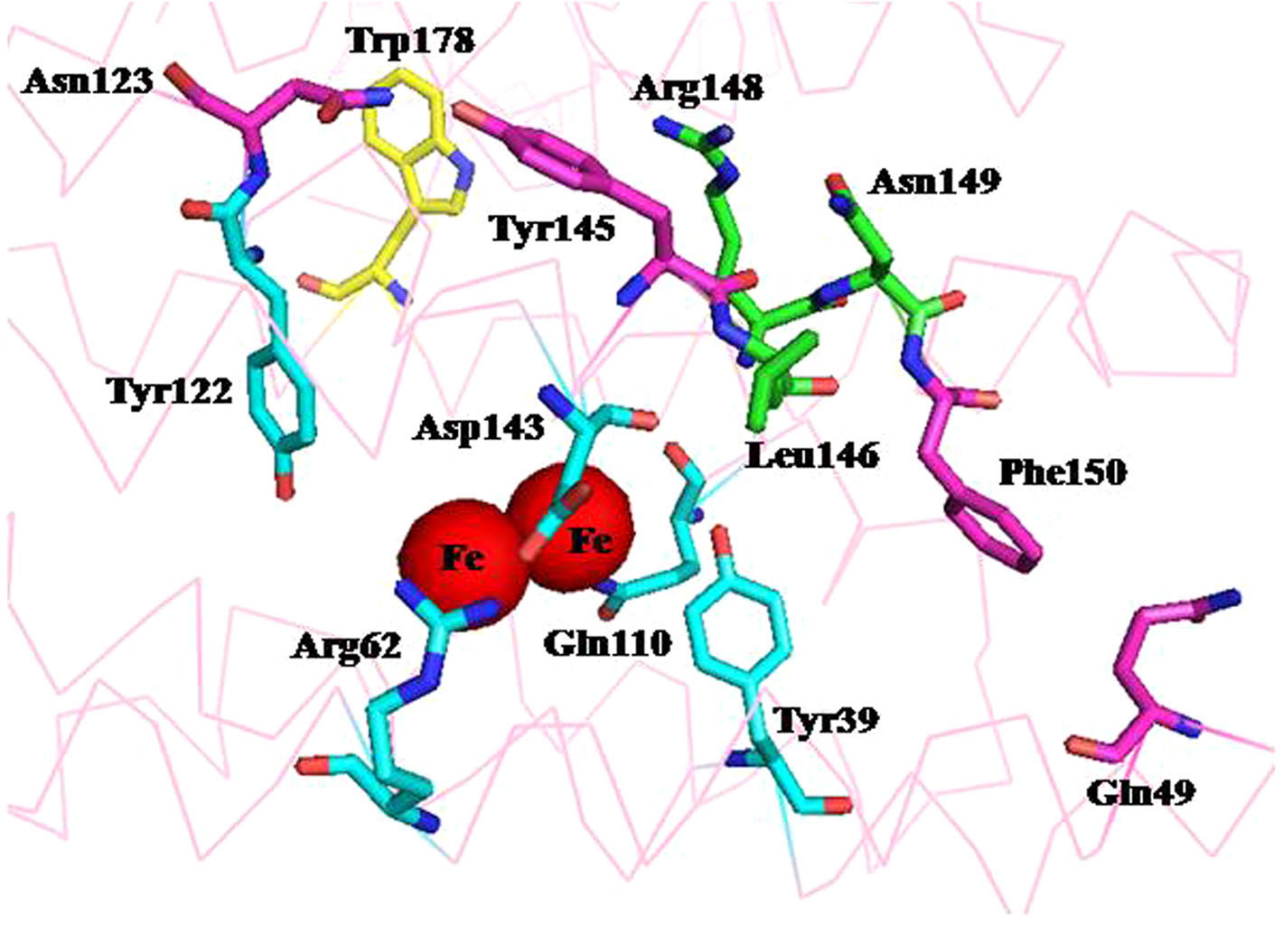
Identified residues based on the crystal structure of ADO from *Synechococcuselongatus* PCC7942 (1593; PDB code:4RC5). The identified residues include those close to the di-iron center (Tyr39, Gln110, Tyr122), the protein surface (Trp178), and involved in the hydrogen-bonding network (Arg62, Asp143) and the oligopeptide whose conformation changed (Leu/Thr146, Leu148, Asn149 and Tyr/Phe150) in the absence of the diiron center

**Fig. 3:**
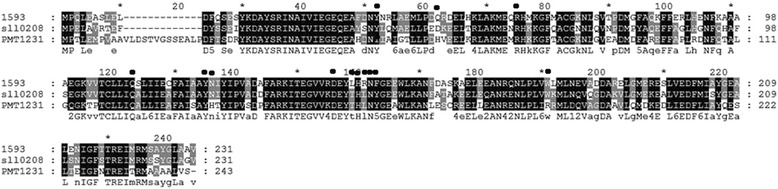
Sequence alignment of 1593, sll0208 and PMT1231. The residues investigated in this paper are labelled with *black dots* above the sequence

Based on the structures of 1593 (cADO from *Synechococcus elongates* PCC7942) (PDB code: 4RC5) and sll0208 (cADO from *Synechocystis* sp. PCC6803) (PDB code: 4Z5S), the overall structures are similar with or without the di-iron center, except the conformation change of the helix H5 in the structure of metal-free cADO [9]. The switch of the helix H5 from helix to loop resulted in conformational changes of a number of amino acids (residues 144 to 150), including the two iron-coordinating residues Glu144 and His147. The observation suggests that the residues involved in the oligopeptide may impact cADO activity. The residues (144 to 150 for 1593) comprising of that oligopeptide are conservable among cADOs (sequence alignment of more than 150 cADOs, which were found by subjecting to a BLAST search of the sequence of 1593) (Additional files 1 and 2) [24]. For example, Glu144 and His147 (1593 used as a reference), two ligands of the di-iron center, are completely conserved. Tyr145 is highly conserved, and is Ser/Ala in several cADOs (Additional files 1 and 2), and is also involved in a hydrogen bond with Asn123 (described in detail in the following part). Residue 146 is variable, and is Leu/Thr/Ser/Glu, etc. Residue 148 is Leu among more than around 140 cADOs, and is Arg in 4 cADOs. Residue Asn149 is highly conservable, and is Asp/Lys in a few cADOs. Tyr150 shows high conservativeness among ~100 cADOs, and is Phe in ~50 cADOs. As mentioned above, the ligands of the di-iron center such as Glu144 and His147 were not investigated. The residues Leu146, Asn149 and Phe150 of 1593, and Thr146, Leu148 and Tyr150 of sll0208, which might have some influence on cADO activity due to the different properties of their side chains, were chosen for mutagenesis (Figs. 2 and 3).

#### Residues involved in two hydrogen bonds

**Fig. 4:**
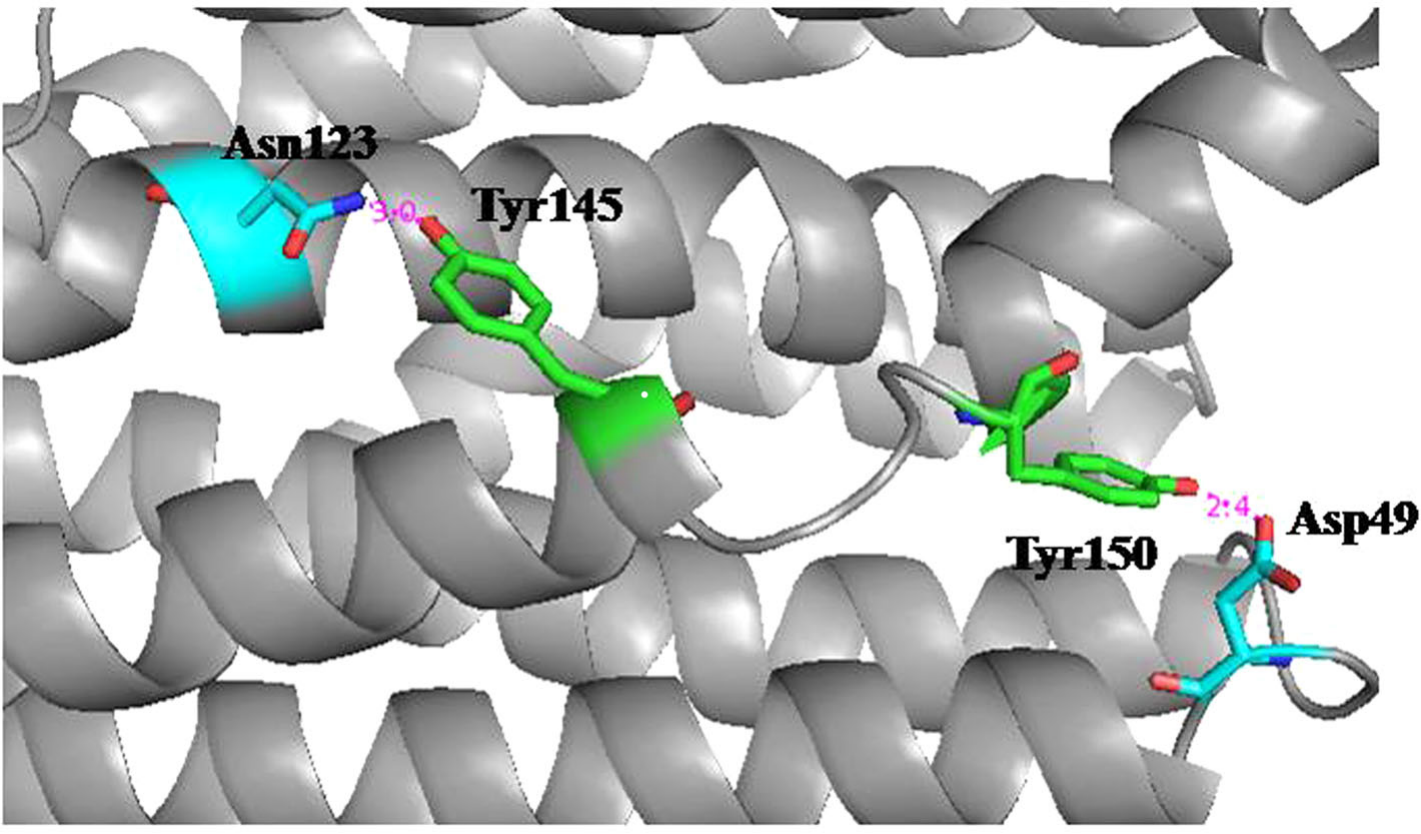
Two hydrogen bonds in sll0208. The hydrogen-bond lengths between Asn123 and Tyr145 and between Tyr150 and Asp49 are 3.0 and 2.4 Å respectively

According to the crystal structures of sll0208 and PMT1231 (ADO from *Prochlorococcus marinus* MIT9313) (PDB code: 2OC5), it was observed that residues Tyr145 and Tyr150 which are equivalent to Tyr158 and Tyr163 of PMT1231 respectively were involved in two hydrogen bonds (between sll0208^Asp49^/PMT1231^His62^ and sll0208^Tyr150^/PMT1231^Tyr163^, and between sll0208^Asn123^/PMT1231^His136^ and sll0208^Tyr145^/PMT1231^Tyr158^) (Fig. 4). However, there is only one hydrogen bond between Asn123 and Tyr145 in 1593, and the corresponding residues forming the other hydrogen bond in sll0208 and PMT1231 are Gln49 and Phe150 in 1593 (Figs. 2 and 3). Residue 49 is variable, and is Gln/Asn/His/Asp/Ser/Glu, etc., all containing the polar side chains (Additional files 1 and 2). Residue Asn123 is conservable among ~120 cADOs, and is His in 32 cADOs. Considering that these three cADOs showed different activities against *n*-hexadecanal (unpublished results), these two hydrogen bonds may have some effects on cADO activity. The residues Gln49, Asn123 of 1593 and Asp49, Asn123 of sll0208 were investigated.

#### Residues close to the di-iron center

The residues around the di-iron center provide conformational constraints that control the geometry of the di-iron center. According to the structure of 1593, residues Tyr39, Arg62, Gln110, Tyr122 and Asp143 are close to the di-iron center, which might interact with the nearby residues including the ligands of di-iron: Tyr39 with Glu60 (ligand), His147 (ligand), Gln110 and Ser111; Arg62 with His147 (ligand) and Asp143; Gln110 with Glu115 (ligand), Asn38 and Tyr39; Tyr122 with Glu32 (ligand), Glu144 (ligand) and Val28 (Fig. 2); Asp143 with Arg62 and His147 (ligand). Residue Tyr39 is highly conserved, and is Phe in 8 cADOs (Additional files 1 and 2). Arg62, Gln110, Tyr122 and Asp143 show high conservativeness among ~150 cADO. Likewise, the residues coordinating the di-iron center like Glu32, Glu44, Glu60, Glu115, Glu144 and His147 were excluded in this study. Residues Tyr39, Arg62, Gln110, Tyr122 and Asp143 of 1593 were selected for investigation.

#### Residues located on the protein surface

Based on sequence alignment of 150 cADOs, Trp178 of 1593 is highly conserved among ~120 cADOs, and is Arg/Lys in ~30 cADOs. The side-chains of Trp and Arg/Lys exhibited completely different properties: hydrophobicity versus hydrophilicity. Moreover, according to the crystal structure of 1593, Trp178 is positioned at Helix 6 and exposed to the protein surface (Fig. 2). These facts prompted us to presume that hydrophobicity or hydrophilicity of this residue might have some influence on cADO activity. Therefore, this residue was studied.

### Site-directed mutagenesis, overexpression and purification

We have observed that 1593 is more active than sll0208 against *n*-hexadecanal, so Thr146, Leu148 and Tyr150 of sll0208 were respectively mutated into the counterparts of 1593 - Leu, Arg and Phe (Fig. 3). For comparison, Leu146 and Phe150 of 1593 were also mutated into the corresponding ones of sll0208 - Thr and Tyr respectively. Since PMT1231 is more active than sll0208 towards *n*-hexadecanal under our assay conditions (unpublished results), Asp49 and Asn123 of sll0208 were mutated into the corresponding residues of PMT1231 - His and His respectively (Fig. 3). In order to investigate the effects of the polar side chains of some residues on cADO activity, the single site-directed mutants Y39F, R62A, Q110L, Y122F, D143A and N149A were constructed for 1593. In addition, N123H and W178R of 1593 were made based on the conservativeness.

### Enzymatic activities of wild-type cADOs and variants

*n*-Hexadecanal and *n*-heptanal were used as the substrates to investigate the effects of mutations on enzymatic activities of 1593 and sll0208. When *n*-hexadecanal was used as a substrate, the yields of *n*-pentadecane were quantified. While *n*-heptanal was used as a substrate, the apparent *k*_*cat*_ values were measured.

Compared with WT (the wild type) 1593, the apparent *k*_*cat*_ values of 1593^W178R^, 1593^Q49H/N123H/F150Y^, 1593^L146T^, 1593^F150Y^ and 1593^Q49H/F150Y^ were enhanced by 226.7 ± 10.3%, 93.3 ± 8.6%, 93.3 ± 8.1%, 68.9 ± 6.2%, and 60 ± 5% respectively (Table 1). 1593^R62A^ exhibited significantly reduced *k*_cat_^app^ value, and dropped by 91.1 ± 7.4% (Table 1). 1593^Y122F^ and 1593^Q110L^ displayed moderate activity, whereas the catalytic activities of 1593^Y39F^, 1593^N123H^, 1593^N149A^ and 1593^D143A^ were reduced by ≤ 31.1 ± 2.6%. The yields of *n*-pentadecane of the enzyme variants showed the same trend as the apparent *k*_*cat*_ values (Table 1).

**Table 1:** Apparent *k*_*cat*_ values and yields of *n*-pentadecane of WT 1593, WT sll0208 and variants. The apparent *k*_*cat*_ values were determined using 2 mM *n*-heptanal as the substrate

		*k*_*cat*_^app^ (Min^−1^)	Yield of *n*-pentadecane (μM)
1593	WT	0.45 ± 0.06	12.1 ± 0.4
	L146T	0.87 ± 0.1	14.2 ± 1.2
	N149A	0.31 ± 0.04	8.7 ± 0.7
	F150Y	0.76 ± 0.08	12.2 ± 0.6
	N123H	0.43 ± 0.05	10.4 ± 0.9
	Q49H/F150Y	0.72 ± 0.07	14.4 ± 0.6
	Q49H/N123H/F150Y	0.87 ± 0.09	15.0 ± 0.1
	Y39F	0.37 ± 0.05	10.4 ± 0.8
	Q110L	0.23 ± 0.03	5.8 ± 0.2
	Y122F	0.15 ± 0.02	2.1 ± 0.04
	R62A	0.04 ± 0.001	0.4 ± 0.06
	D143A	0.36 ± 0.05	9.2 ± 0.2
	W178R	1.47 ± 0.1	17.4 ± 0.2
sll0208	WT	0.44 ± 0.05	1.4 ± 0.1
	T146L	0.17 ± 0.02	1.4 ± 0.1
	L148R	0.75 ± 0.08	5.1 ± 0.2
	Y150F	0.42 ± 0.04	2.2 ± 0.1
	D49H	0.22 ± 0.03	1.8 ± 0.1
	N123H	0.14 ± 0.02	1.0 ± 0.08
	D49H/N123H	0.73 ± 0.08	3.4 ± 0.3

The yield of *n*-pentadecane was determined using 150 μM using *n*-hxadecanal as the substrate

Compared with WT sll0208, the apparent *k*_*cat*_ values of sll0208^L148R^ and sll0208^D49H/N123H^ were increased by 70.5 ± 5.3% and 65.9 ± 4.4% respectively, whereas those of sll0208^D49H^, sll0208^N123H^ and sll0208^T146L^ were significantly reduced (Table 1). The activity of sll0208^Y150F^ was not almost affected. The yields of *n*-pentadecane of the enzyme variants demonstrated similar trend to the apparent *k*_*cat*_ values (Table 1).

The kinetic parameters towards *n*-heptanal were also determined for some variants showing higher activity than WT. The variants L148R and D49H/N123H of sll0208, and W178R, Q49H/N123H/F150Y, L146T and Q49H/F150Y of 1593 showed higher *k*_*cat*_ values than WT and comparable *K*_*m*_ values to WT, indicating that these mutations had significant effects on activity, but no big impact on substrate binding (Table 2). The observation that the *k*_*cat*_ value of F150Y was higher than that of WT 1593 and its *K*_*m*_ value was much lower than that of WT 1593 suggested that replacement of phenylalanine 150 with tyrosine not only affected activity but also substrate binding (Table 2).

**Table 2:** Kinetic parameters of WT 1593, WT sll0208 and some variants

		*K*_*m*_ (mM)	*k*_*cat*_ (min^−1^)	*k*_*cat*_/*K*_*m*_ (min^−1^mM^−1^)
1593	WT [17	0.30 ± 0.02	0.48 ± 0.01	1.6 ± 0.2
	L146T	0.34 ± 0.08	0.94 ± 0.08	2.76 ± 0.3
	F150Y	0.20 ± 0.04	0.75 ± 0.04	3.75 ± 0.4
	Q49H/F150Y	0.33 ± 0.05	0.87 ± 0.04	2.64 ± 0.2
	Q49H/N123H/F150Y	0.35 ± 0.02	1.04 ± 0.02	2.97 ± 0.3
	W178R	0.34 ± 0.08	1.81 ± 0.15	5.32 ± 0.4
sll0208	WT	0.35 ± 0.07	0.59 ± 0.04	1.69 ± 0.2
	L148R	0.32 ± 0.08	0.91 ± 0.08	2.84 ± 0.3
	D49H/N123H	0.38 ± 0.08	0.83 ± 0.08	2.18 ± 0.2

The kinetic parameters against *n*-heptanal were determined

Interestingly, the *k*_*cat*_ values of 1593^W178R^, 1593^Q49H/N123H/F150Y^ and 1593^L146T^, close to or above 1 min^−1^, were obtained. Especially, 1593^W178R^ showed 3.8-fold improved *k*_*cat*_ value than WT 1593, and exhibited the highest catalytic efficiency (*k*_cat_/*K*_m_) among all variants, 3.3-fold higher than WT (Table 2).

## Discussion

Although the conditions of the cADO-catalyzed reaction have been optimized through different attempts, the turnover numbers are still below ~1 min^−1^ [8, 14–17, 22]. Therefore, the sluggish kinetics of cADO has become a bottle-neck for biofuel production in cyanobacteria, and to identify the activity related key residues for cADO is definitely required. Since it was observed that cADO-1593 showed the highest activity under our assay conditions among cADOs tested (unpublished results), it was selected as the test sequence for BLAST. In the current paper, we have identified some amino acid residues which impact the enzyme activity of cADO through structure and sequence analysis of some cADOs.

Firstly, the results of 1593^L146T^, 1593^F150Y^, sll0208^T146L^ and sll0208^L148R^ have clearly demonstrated that the side chains of the residues consisting of the oligopeptide (residues 144 to 150 for 1593) whose conformation changed in the absence of the di-iron center had important effects on cADO activity. Crystal structures reveal that these polar residues form important hydrogen bond interactions with residues from other helices, which may contribute to the local structural stability of the oligopeptide. In addition, the polar side chains of these residues might interact with the nearby residues, for example, in 1593 (Protein code: 4RC5) Arg148 with Asn149 and Glu152, Asn149 with Arg148 and Glu152; in sll0208 (Protein code: 4Z5S) Thr146 with Asp143 and Asn149, Asn149 with Thr146 and Glu152, Tyr150 with Gln49; in PMT1231 (Protein code: 2OC5) Thr159 with Asp156 and Asn162, Asn162 with Thr159 and Glu165, Tyr163 with His62, which are equivalent to the residues 146, 143, 149, 152, 150 and 49 of 1593 and sll0208 respectively. Thr159 of PMT1231 and the equivalent one (Thr146) of sll0208 are also involved in the hydrogen-bonding network close to the di-iron center. Replacement of the residues containing the polar side chains with ones containing the nonpolar side chains led to removal of the possible interaction with nearby residues, which might have severe influence on protein folding, stability, even activity. In contrast, substitution of amino acids with the nonpolar side chains by ones with the polar side chains gave the reverse impact, as observed for 1593^L146T^, sll0208^L148R^ and 1593^F150Y^. The enhanced activities of these three variants are presumably due to local stabilization caused by newly established interaction of three residues with the nearby amino acids.

Secondly, based on the structures of sll0208 and PMT1231, a hydrogen bond is possibly established between Tyr150 and Gln49 in 1593 while Phe150 of 1593 was substituted by Tyr. Activities of both F150Y and Q49H/F150Y of 1593 were enhanced, suggesting that the hydrogen bond between these two residues is important for 1593. The triple mutant 1593^Q49H/F150Y/N123H^ showed higher activity than WT 1593, 1593^Q49H/F150Y^ and 1593^F150Y^, and the double mutant sll0208^D49H/N123H^ exhibited improved activity than WT sll0208, sll0208^D49H^ and sll0208^N123H^. Thus, some synergistic effects were observed for 1593^Q49H/F150Y/N123H^ and sll0208^D49H/N123H^, implying that these two hydrogen bonds are beneficial for cADO activity and the co-existence of two His in both hydrogen bonds is more important than the presence of only one His. Unexpectedly, the Y150F substitution of sll0208, in which the hydrogen bond between Tyr150 and Asp49 is removed, had negligible effect on the catalytic activity.

Thirdly, replacement of the residues Arg62, Gln110 and, Tyr122 of 1593 having the polar side chains by those containing the nonpolar ones resulted in significantly decreased activity, which could be possibly due to removal of the interaction with the nearby residues. These results confirmed that the residues close to the di-iron center impacted cADO activity and their polar side chains are important for cADO.

**Fig. 5:**
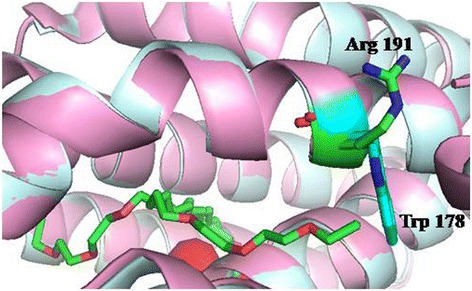
Structural superimposition of L194A of PMT1231 (palecyan, PDB code: 4PGI) and 1593 (*light pink*, PDB code: 4QUW). Arg191 of PMT1231, Trp178 of 1593 and two substrate-binding modes were shown

Finally, when the highly conserved residue Trp178 of 1593 was mutated into Arg, the W178Rvariant showed the highest cADO activity by far. Trp178 of 1593 and the equivalent one (Arg191) of PMT1231 are rightly located on the protein surface according to the crystal structures of cADOs (PDB codes: 2OC5, 4KVQ, 4KVR and 4KVS). The enhanced activity of W178R could be possibly due to increased protein stability resulting from mutation of exposed hydrophobic amino acids into hydrophilic ones such as arginine or the change of hydrophobicity into hydrophilicity of Trp178 exposed to the solvent at the protein surface contributes to cADO activity [25]. Moreover, according to the crystal structure of the mutant L194A of PMT1231, the substrate might enter the protein at Leu194. This mode of substrate binding is different from previously determined cADO structures with long-chain fatty acids bound [8]. In this structure, the side-chain of Arg191, equivalent to Trp178 of 1593, points away from the substrate, which may be beneficial for substrate entry (Fig. 5). However, according to the structure of 1593 (PDB code: 4QUW), the side-chain of Trp178 points towards the substrate, which might interfere with substrate entry (Fig. 5). Thus, mutation of Trp178 into Arg might contribute to substrate entry and binding, leading to enhanced activity observed for W178R.

## Conclusions

Some amino acids which could affect cADO activity were identified based on the crystal structures and sequence alignment of cADOs. The residues close to the di-iron center, the protein surface, and those involved in the hydrogen-bonding network and the oligopeptide whose conformation changed, exerted great influence on cADO activity. Several mutations led to significantly decreased activity. Some enzyme variants showed improved activity than the wild type, and the *k*_*cat*_ values of several of them close to or above 1 min^−1^ were achieved for the first time in particular. We identified some important residues for the catalytic activity of cADO. The study would be helpful for establishing an efficient cell factory of biofuel production in cyanobacteria.

## Methods

### Materials

Chemicals were from Sigma, Merck or Ameresco. Oligonucleotides and the gene encoding ADO (aldehyde-deformylatingoxygenase) Synpcc7942_1593 from *Synechococcus elongates* PCC7942 with codon optimization were synthesized by Shanghai Sangon Biotech Co. Ltd (China) [16]. *Pfu* DNA polymerase, restriction endonucleases *Eco*RI and *Not*I were from Fermentas or Takara Biotechnology. *Dpn*I was from New England BioLabs. The kits used for molecular cloning were from Omega Bio-tek or Takara Biotechnology. Nickel column and expression vector pET-28a(+) were from Novagen. Amicon YM10 membrane was from Millipore.

### Bacterial strains, plasmids, and media

*E. coli* DH5α was used for routine DNA transformation and plasmid isolation. *E. coli* BL21(DE3) was utilized for overexpression of cADO. *E. coli* strains were routinely grown in Luria-Bertani broth at 37 °C with aeration or on LB supplemented with 1.5% (w/v) agar. 100 μg/ml Kanamycin was added when required.

### DNA manipulations

General molecular biology techniques were carried out by standard procedures [26]. DNA fragments were purified from agarose gels using the DNA gel extraction kit. Plasmid DNA was isolated using the plasmid miniprep kit.

The gene *sll0208* encoding ADO from *Synechocystis* sp. PCC6803 (sll0208) was amplified with the forward primer (5′- GCCTTACATATGATGCCCGAGCTTGCTG-3′, the *Nde*I I restriction site underlined) and the reverse primer (5′- CAACTACTCGAGCTAGACTCCGGCCAAACC-3′, the *Xho*I restriction site underlined) using genomic DNA as a template. The PCR products were recovered, and digested with restriction enzymes *Nde*I and *Xho*I respectively, and re-cloned into the vector pET-28a(+) digested with *Nde*I and *Xho*I, respectively.

### Construction of site-directed mutants

Site-directed mutants were constructed according to the standard QuikChange Site-Directed Mutagenesis protocol (Stratagene Ltd) using wild-type (WT) 1593 or sll0208 as templates and the primers listed in Table S1 (Additional file 3).

For construction of double and triple mutants, WT 1593 or sll0208 harboring single or double mutation(s) was used as a template respectively following the same protocol as above.

### Protein overexpression and purification

WT cADOs and variants were overexpressed in *E. coli* BL21(DE3) and purified on Nickel column as reported [16]. The purity of protein was checked by SDS-PAGE (Additional file 4). Apo-ADO was prepared according to the published procedure, and the diferrous form of ADO was reconstituted by the addition of the stoichiometric amounts of ferrous ammonium sulfate to the apo-ADO prior to assay [14–16]. Proteins were concentrated with Amicon YM10 membrane (10 kDa cut-off). The protein concentration was determined by the Bradford method using bovine serum albumin as a standard.

### Enzyme activity assay

All enzymatic assays were carried out in triplicate. According to the published procedure [16, 17], assays were carried out in HEPES buffer, containing 100 mM KCl and 100 mM HEPES, pH 7.2. The reaction mixtures contain NADH (750 μM), catalase (1 mg/ml), ferrous ammonium sulfate (80 μM), PMS (Phenazine methosulfate) (75 μM), appropriate amount of aldhydes (150 μM for *n*-hxadecanal, 2 mM for *n*-heptanal), cADO (20 μM for *n*-hxadecanal, 5 μM for *n*-heptanal). When *n*-hxadecanal was used as the substrate, the yields of *n*-pentadecane were quantified by GC-MS. While *n*-heptanal was used as substrate, the apparent *k*_*cat*_ values (2 mM of *n*-heptanal was utilized) were measured. The control using *E. col*i containing the vector pET-28a only didn’t show any ADO activity (data not shown).

*K*_*m*_ and *V*_*max*_ values of WT 1593, WT sll0208 and some variants against *n*-heptanal were determined according to the Michaelis-Menten equation of GraphPad Prism 5 (Additional file 5). The *k*_*cat*_ values were calculated from *V*_*max*_ on the basis of the molecular weight of enzymes.

## Additional files

Additional file 1: Sequence alignment of cADOs (1–100). (DOCX 24 kb)
Additional file 2: Sequence alignment of cADOs (101–150). (DOCX 20 kb)
Additional file 3: Table S2. Primers used for construction of site-directed mutants. (DOCX 16 kb)
Additional file 4: SDS PAGE analysis of WT 1593, WT sll0208 and all variants. (DOCX 991 kb)
Additional file 5: Original data for determination of the kinetic parameters. (DOCX 367 kb)

## Abbreviations

ACP: Acyl carrier protein
ADO: Aldehyde-deformylating oxygenase
cADO: Cyanobacterial aldehyde-deformylating oxygenase
Fd: Ferredoxin
FNR: Ferredoxin-NADP^+^ reductase
PMS: Phenazine methosulfate
