# Supplementary material for: Identification of residues important for the activity of aldehyde-deformylating oxygenase through investigation into the structure-activity relationship
Source: BMC Biotechnol. 2017 Mar 16;17:31. doi: 10.1186/s12896-017-0351-8 (PMC5356278; doi:10.1186/s12896-017-0351-8)
Supplement: Additional file 1: — Sequence alignment of cADOs (1–100). (DOCX 24 kb) [file 12896_2017_351_MOESM1_ESM.docx]

Additional file 1 – Sequence alignment of cADOs (1-100)：

SP|Q54764|ALDEC_SYNE7 -----------------------MPQL--------------------EASLELDFQSESY 17

TR|Q5N627|Q5N627_SYNP6 MRTPWDPPNPTFSLSSVSGDRRLMPQL--------------------EASLELDFQSESY 40

TR|Q8KPT4|Q8KPT4_SYNE7 MRTPWDPPNPTFSLSSVSGDRRLMPQL--------------------EASLELDFQSESY 40

TR|U3M3N6|U3M3N6_9CYAN ------------------------------------------------------------

TR|K9S7C1|K9S7C1_9CYAN -----------------------MPQL--------------------EASLELDFHSETY 17

TR|D5A1Z8|D5A1Z8_ARTPN -----------------------MPQL-------------------ETI-AELDFQNETY 17

TR|A0A067RRZ6|A0A067RRZ6_ARTPT -----------------------MPQL-------------------ETI-AELDFQNETY 17

TR|U7QM55|U7QM55_9CYAN -----------------------MPQL-------------------EAI-AEIDFNTNTY 17

TR|K1WZT6|K1WZT6_ARTPT -----------------------MPQL-------------------ETI-TELDFQNETY 17

TR|H1WAD2|H1WAD2_9CYAN -----------------------MPQL-------------------ETI-TELDFQNETY 17

TR|W6SNU7|W6SNU7_9CYAN -----------------------MPQL-------------------ETI-TELDFQNETY 17

TR|B5W0S5|B5W0S5_ARTMA -----------------------MPQL-------------------ETI-TELDFQNETY 17

TR|W5U411|W5U411_9CYAN -----------------------MPQL--------------------EASLALDFQSEAY 17

TR|A0A084YC72|A0A084YC72_9CYAN -----------------------MQQL--------------------AAQPEIDFHSDSY 17

TR|U9WCI1|U9WCI1_9CYAN -----------------------MPQL--------------------EASPTLDFQSEAY 17

TR|K9WII2|K9WII2_9CYAN -----------------------MQQL--------------------AVSPEFDFTSESY 17

TR|A0A073CMN4|A0A073CMN4_PLAAG --------------------------------------------MPQVEAITIDFQSETY 16

TR|K8GFP8|K8GFP8_9CYAN -----------------------MPQL--------------------EASVAIDFRSEAY 17

TR|D8FVZ2|D8FVZ2_9CYAN -----------------------MQQL--------------------EASPAIDFETATY 17

TR|B4VSW8|B4VSW8_9CYAN ---------------MQTGENLLMQQL--------------------TVSQELDFNSETY 25

TR|W5U332|W5U332_PLAAG --------------------------------------------MPQVEAITIDFQSETY 16

TR|K9V615|K9V615_9CYAN -----------------------MQQL--------------------EAELKIDFNSEIY 17

TR|K9F0G4|K9F0G4_9CYAN -----------------------MPQL--------------------EASPALDFQSEAY 17

TR|A0YKK4|A0YKK4_LYNSP -----------------------MPQL-------------------EAI-AEIDFNTNTY 17

TR|I4I7T6|I4I7T6_9CHRO -------------------------------------------MPELAVPLELDFTSETY 17

TR|I4FNH9|I4FNH9_MICAE -------------------------------------------MPELAVPLELDFTSETY 17

TR|H2DDT7|H2DDT7_NOSS6 -----------------------MQPL--------------------AAELEIDFQSETY 17

TR|G9HTG5|G9HTG5_9NOSO -----------------------MQPL--------------------AAELEIDFQSETY 17

TR|I4GYG9|I4GYG9_MICAE -------------------------------------------MPELAVPLELDFTSETY 17

TR|A0Z9X5|A0Z9X5_NODSP -----------------------MQQL--------------------AAELKIDFQSEKY 17

TR|K9SPY2|K9SPY2_9CYAN ----------------------MIGQQ-------------------LDIVTELDYNSDTY 19

TR|B0JY93|B0JY93_MICAN -------------------------------------------MPELAVPLELDFTSETY 17

TR|I4H417|I4H417_MICAE -------------------------------------------MPELAVPLELDFTSETY 17

TR|I4FY74|I4FY74_MICAE -------------------------------------------MPELAVPLELDFTSETY 17

TR|I4HKI6|I4HKI6_MICAE -------------------------------------------MPELAVPLELDFTSETY 17

TR|F5UGX9|F5UGX9_9CYAN -----------------------MPQL--------------------EASPTIDFQTETY 17

TR|L7E3D3|L7E3D3_MICAE -------------------------------------------MPELAVPLELDFTSETY 17

TR|K9VE50|K9VE50_9CYAN -----------------------MPQL--------------------EASPTIDFQTETY 17

TR|I4IM50|I4IM50_MICAE -------------------------------------------MPELAVSSELDFTSETY 17

TR|K9QA48|K9QA48_9NOSO -----------------------MQQL--------------------AAELEIDFQSETY 17

TR|I4FD05|I4FD05_MICAE -------------------------------------------MPELAVPLELDFTSETY 17

TR|K9Y9U4|K9Y9U4_HALP7 -------------------------------------------MQDVAVTPTLDYNSETY 17

TR|A8YJD6|A8YJD6_MICAE -------------------------------------------MPELAVPLELDFTSETY 17

TR|L8NPI8|L8NPI8_MICAE -------------------------------------------MPELAVPLELDFTSETY 17

TR|S3JGS6|S3JGS6_MICAE -------------------------------------------MPELAVPLELDFTSETY 17

TR|I4HLI6|I4HLI6_MICAE -------------------------------------------MPELAVPLELDFTSETY 17

TR|K9QXA2|K9QXA2_NOSS7 -----------------------MQQI--------------------AGDLEIDFKSEKY 17

TR|Q8YLL5|Q8YLL5_NOSS1 -----------------------MQQV--------------------AADLEIDFKSEKY 17

TR|K9TGS5|K9TGS5_9CYAN -----------------------MPQL-------------------EATSATLDFQSPTY 18

TR|Q3MA38|Q3MA38_ANAVT -----------------------MQQV--------------------AADLEIDFKSEKY 17

TR|K9W4T2|K9W4T2_9CYAN -----------------------MAQI----------------------AASIDFQSETY 15

TR|G5J0P0|G5J0P0_CROWT -------------------------------------------MQELAVRSELDFNSETY 17

TR|Q4C7T3|Q4C7T3_CROWT -------------------------------------------MQELAVRSELDFNSETY 17

TR|I4GKL8|I4GKL8_MICAE -------------------------------------------MPELAVPLELDFTSETY 17

TR|T2IH71|T2IH71_CROWT -------------------------------------------MQELAVRSELDFNSETY 17

TR|T2JF13|T2JF13_CROWT -------------------------------------------MQELAVRSELDFNSETY 17

TR|K9TXF6|K9TXF6_9CYAN -----------------------MQQV--------------------AATSEIDFHSETY 17

TR|K9WRL8|K9WRL8_9NOST -----------------------MQQI-------------------ADQVEKMDFQSETY 18

TR|G6FV22|G6FV22_9CYAN -----------------------MQQL--------------------ADQPEIDFHSETY 17

TR|K9X9I4|K9X9I4_9CHRO ----------------------MQQLA--------------------ATAAELDFHSETY 18

TR|K9ZJT3|K9ZJT3_ANACC -----------------------MQQL-------------------VEQIEKIDFQSEEY 18

TR|B4WJ48|B4WJ48_9SYNE -----------------------MQTL--------------------EVSPAMDFQSETY 17

TR|W5U379|W5U379_9CYAN -------------MYHIFRSGRTMQQL--------------------ANQPDIDFKSETY 27

TR|K9YV86|K9YV86_DACSA -------------------------------------------MQDVAVTPTLDYTSETY 17

TR|W5U417|W5U417_9CYAN -------------MYHIFRSGRTMQQL--------------------ANQPDIDFKSETY 27

TR|A0A068MTA8|A0A068MTA8_SYNY4 -------------------------------------------MPELAVRTEFDYSSETY 17

TR|Q112R8|Q112R8_TRIEI -----------------------MPKLEIIPTMDSQSETKLEKVKSQSEGDQINFETETY 37

TR|D7E2W1|D7E2W1_NOSA0 -----------------------MQQL-------------------VEEIEKIDFQSEKY 18

TR|B1WR71|B1WR71_CYAA5 -------------------------------------------MQELALRSELDFNSETY 17

TR|F7US88|F7US88_SYNYG -------------------------------------------MPELAVRTEFDYSSEIY 17

TR|H0PB35|H0PB35_9SYNC -------------------------------------------MPELAVRTEFDYSSEIY 17

TR|H0NYN3|H0NYN3_9SYNC -------------------------------------------MPELAVRTEFDYSSEIY 17

TR|L8AP37|L8AP37_BACIU -------------------------------------------MPELAVRTEFDYSSEIY 17

TR|M1LIQ1|M1LIQ1_9SYNC -------------------------------------------MPELAVRTEFDYSSEIY 17

TR|H0PG18|H0PG18_9SYNC -------------------------------------------MPELAVRTEFDYSSEIY 17

TR|K9PTP6|K9PTP6_9CYAN -----------------------MQQL-------------------TEQ-SEIDFQSEIY 17

TR|X5JQD7|X5JQD7_9NOST -----------------------MQQL--------------------ATQSEIDFSSEIY 17

SP|Q55688|ALDEC_SYNY3 -------------------------------------------MPELAVRTEFDYSSEIY 17

TR|K9ULL9|K9ULL9_9CHRO -----------------------MVQM--------------------AASLELNFTSEIY 17

TR|K7WTY7|K7WTY7_9NOST -----------------------MQQL-------------------VDQIEKIDFQSKIY 18

TR|K9Z5G1|K9Z5G1_CYAAP -------------------------------------------MQQ-LVEPTIDFNSDVY 16

TR|A3INE1|A3INE1_9CHRO -------------------------------------------MQELALRSELDFNSETY 17

TR|D4TRY1|D4TRY1_9NOST -----------------------MQQL-------------------VEQIEKTDFQSAKY 18

TR|V5V382|V5V382_9CHRO -------------------------------------------MTTATATPDLDYHSDRY 17

TR|B8HSZ3|B8HSZ3_CYAP4 -------------------------------------------MPQVQSPSAIDFYSETY 17

TR|M1X0T0|M1X0T0_9NOST ---------------------MTMQQL--------------------ATQSEIDFSSEIY 19

SP|B2J1M1|ALDEC_NOSP7 -----------------------MQQL-------------------TDQSKELDFKSETY 18

TR|L8N0G8|L8N0G8_9CYAN -------------------------------------------MGQTLEAVAIDYQTENY 17

TR|M1WVU9|M1WVU9_9NOST -----------------------MQQL--------------------ATQSEIDFSSEIY 17

TR|B0C9L0|B0C9L0_ACAM1 -------------------------------------------MPQTQAISEIDFYSDTY 17

TR|U3M1Y7|U3M1Y7_SYNEL ------------------------------------------------------------

TR|K9RFH5|K9RFH5_9CYAN -----------------------MQEL--------------------ISPSELDYRSETY 17

TR|L8LLH3|L8LLH3_9CHRO -------------------------------------------MQELTMPRELDYQSPEY 17

TR|A0A077JJ22|A0A077JJ22_9CYAN -------------------------------------------MQELATRSELEFNSETY 17

TR|B7JUH1|B7JUH1_CYAP8 -------------------------------------------MQELVQRSELDFTNPTY 17

TR|C7QUV0|C7QUV0_CYAP0 -------------------------------------------MQELVQRSELDFTNPTY 17

TR|U3M0X3|U3M0X3_ARTPT ------------------------------------------------------------

TR|D4TEY2|D4TEY2_9NOST -----------------------MQQL-------------------VEQIEKTGFQSAKY 18

TR|D3EPA5|D3EPA5_ATETH -------------------------------------------MQELALRSELDFNSETY 17

TR|A0A086CFQ6|A0A086CFQ6_ATETH -------------------------------------------MQELALRSELDFNNETY 17

SP|Q54764|ALDEC_SYNE7 KDAYSRINAIVIEGEQEAFDNYNRLAEMLPDQRDELHKLAKMEQRHMKGFMACGKNLSVT 77

TR|Q5N627|Q5N627_SYNP6 KDAYSRINAIVIEGEQEAFDNYNRLAEMLPDQRDELHKLAKMEQRHMKGFMACGKNLSVT 100

TR|Q8KPT4|Q8KPT4_SYNE7 KDAYSRINAIVIEGEQEAFDNYNRLAEMLPDQRDELHKLAKMEQRHMKGFMACGKNLSVT 100

TR|U3M3N6|U3M3N6_9CYAN --------AIVIEGEQEAFDNYNRLAEMLPDQRDELHKLAKMEQRHMKGFMACGKNLSVT 52

TR|K9S7C1|K9S7C1_9CYAN KDAYSRINAIVIEGEQEAYENYLKLAELLPDNQDELVRLSKMESRHKKGFQACGRNLEVT 77

TR|D5A1Z8|D5A1Z8_ARTPN KDAYSRINAIVIEGEQEAYDNYIKLGEMLPEEREELIRLSKMEKRHMKGFQACGRNLEVS 77

TR|A0A067RRZ6|A0A067RRZ6_ARTPT KDAYSRINAIVIEGEQEASDNYIKLGEMLPEEREELIRLSKMEKRHMKGFQACGRNLEVS 77

TR|U7QM55|U7QM55_9CYAN KDAYSRINAIVIEGEQEAHDNYIKLGEMLPHEKDELVRLSKMEKRHMKGFQACGRNLEVT 77

TR|K1WZT6|K1WZT6_ARTPT KDAYSRINAIVIEGEQEASDNYIKLGEMLPEEREELIRLSKMEKRHKKGFQACGRNLEVT 77

TR|H1WAD2|H1WAD2_9CYAN KDAYSRINAIVIEGEQEASDNYIKLGEMLPEEREELIRLSKMEKRHKKGFQACGRNLEVT 77

TR|W6SNU7|W6SNU7_9CYAN KDAYSRINAIVIEGEQEASDNYIKLGEMLPEEREELIRLSKMEKRHKKGFQACGRNLEVT 77

TR|B5W0S5|B5W0S5_ARTMA KDAYSRINAIVIEGEQEASDNYIKLGEMLPEEREELIRLSKMEKRHKKGFQACGRNLEVT 77

TR|W5U411|W5U411_9CYAN KDAYSRINAIVIEGEWEANDNYQTLAEHLSDHKDELIKLARMEKRHMKGFQACGKNLNVT 77

TR|A0A084YC72|A0A084YC72_9CYAN KDAYSRVNAIVIEGEQEAYENYLRLAELLPDNKDDLIRLSKMENRHKKGFEACGRNLQVT 77

TR|U9WCI1|U9WCI1_9CYAN KDAYSRINAIVIEGELEANDNYKKLAENLNEHKDELIKLARMENRHMKGFQACGKNLNVT 77

TR|K9WII2|K9WII2_9CYAN KDAYSRINAIVIEGEQEAHDNYITLAQLLPDHKDELQRLSKMESRHMKGFQACGRNLSVT 77

TR|A0A073CMN4|A0A073CMN4_PLAAG KDAYSRINAIVIEGEQEAYDNYIKLGAMLPEQQENLAGLAKMEMSHKRGFQSCGRNLTVT 76

TR|K8GFP8|K8GFP8_9CYAN RDAYSRINAIVIEGEQEANENYVKLAELLPDSKEQLLGLAKMESRHKKGFEACGRNLEVT 77

TR|D8FVZ2|D8FVZ2_9CYAN KDAYSRINAIVIEGEQEAYDNYIRLGEMLPDQKDVLIALSKMENRHMKGFQACGRNLKVT 77

TR|B4VSW8|B4VSW8_9CYAN KDAYSRINAIVIEGEQEAHQNYIQLAELLPDQKDELTSLAKMENRHKKGFQACGRNLSVT 85

TR|W5U332|W5U332_PLAAG KDAYSRINAIVIEGEQEAYDNYIKLGAMLPEQQENLAGLAKMEMSHKRGFQSCGRNLTVT 76

TR|K9V615|K9V615_9CYAN KDAYSRINAIVIEGEQEAHENYISLGELLPDSKDELVRLSKMESRHKKGFEACGRNLKVT 77

TR|K9F0G4|K9F0G4_9CYAN KDAYSRINAIVIEGEWEANDNYQKLAEHLGEHKDELIKLARMEKRHMKGFQACGKNLNVT 77

TR|A0YKK4|A0YKK4_LYNSP KDAYSRINAIVIEGEQVAHDNYIKLGEMLPDQKDELVRLSKMEKRHMKGFQACGRNLEVT 77

TR|I4I7T6|I4I7T6_9CHRO KSAYSRINAIVIEGEYEANSNYIQLADILADNKEELHRLAKMENRHMKGFQACGQNLQIT 77

TR|I4FNH9|I4FNH9_MICAE KSAYSRINAIVIEGEYEANSNYIQLADILTDNKEELHRLAKMENRHMKGFQACGRNLQIT 77

TR|H2DDT7|H2DDT7_NOSS6 KDAYSRINAIVIEGEQEAYENYIKLAELLPANHDQLIGLSKMESRHRKGFEACGRNLKVT 77

TR|G9HTG5|G9HTG5_9NOSO KDAYSRINAIVIEGEQEAYENYIKLAELLPANHDQLIGLSKMESRHKKGFEACGRNLKVT 77

TR|I4GYG9|I4GYG9_MICAE KSAYSRINAIVIEGEYEANSNYIQLADILTDNKEELHRLAKMENRHMKGFQACGQNLQIT 77

TR|A0Z9X5|A0Z9X5_NODSP KDAYSRINAIVIEGEQEAHDNYITLGEMLPELKDELIRLSKMESRHKKGFEACGRNLSVK 77

TR|K9SPY2|K9SPY2_9CYAN RDAYSRINAIVIEGEQEAYSNYLRLGEMLPDLKDDLAKLAKMEMRHKKGFTACGKNLSVT 79

TR|B0JY93|B0JY93_MICAN KSAYSRINAIVIEGEYEANSNYIQLADILTDNKEELHRLAKMENRHMKGFQACGQNLKIT 77

TR|I4H417|I4H417_MICAE KSAYSRINAIVIEGEYEANSNYIQLADILTDKKEELHRLAKMENRHMKGFQACGRNLQIT 77

TR|I4FY74|I4FY74_MICAE KSAYSRINAIVIEGEYEANSNYIQLADILTDNKEELHRLAKMENRHMKGFQACGRNLQIT 77

TR|I4HKI6|I4HKI6_MICAE KSAYSRINAIVIEGEYEANSNYIQLADILTDNKEELHRLAKMENRHMKGFQACGQNLKIT 77

TR|F5UGX9|F5UGX9_9CYAN KDAYSRINAIVIEGEQEAHDNYLTLAELLADKKAELIGLSKMENRHMKGFQACGRNLKVT 77

TR|L7E3D3|L7E3D3_MICAE KSAYSRINAIVIEGEYEANSNYIQLADILTDNKEELHRLAKMENRHMKGFQACGQNLQIT 77

TR|K9VE50|K9VE50_9CYAN KDAYSRINAIVIEGEQEAHDNYLTLAELLADKKAELVGLSKMENRHMKGFQACGRNLKVT 77

TR|I4IM50|I4IM50_MICAE KSAYSRINAIVIEGEYEANSNYIQLADILTDNKEELHRLAKMENRHMKGFQACGQNLQIT 77

TR|K9QA48|K9QA48_9NOSO KDAYSRINAIVIEGEQEAYENYIKLAELLPANHDQLIGLSKMESRHKKGFEACGRNLKVT 77

TR|I4FD05|I4FD05_MICAE KSAYSRINAIVIEGEYEANSNYIQLADILTDNKEELHRLAKMENRHMKGFQACGQNLQIT 77

TR|K9Y9U4|K9Y9U4_HALP7 KDAYSRINAIVIEGEQEAKDNYLHLIELLPDSKEELTRLSKMEARHKKGFQACGKNLNVT 77

TR|A8YJD6|A8YJD6_MICAE KSAYSRINAIVIEGEYEANSNYIQLADILTDNKEELHRLAKMENRHMKGFQACGQNLQIT 77

TR|L8NPI8|L8NPI8_MICAE KSAYSRINAIVIEGEYEANSNYIQLADILTDNKEELHRLAKMENRHMKGFQACGQNLQIT 77

TR|S3JGS6|S3JGS6_MICAE KSAYSRINAIVIEGEYEANSNYIQLADILTDNKEELHRLAKMENRHMKGFQACGQNLKIT 77

TR|I4HLI6|I4HLI6_MICAE KSAYSRINAIVIEGEYEANSNYIQLADILTDNKEELHRLAKMENRHMKGFQACGQNLKIT 77

TR|K9QXA2|K9QXA2_NOSS7 KDAYSRINAIVIEGEQEAYDNYIQLAEMLPEHKDELIRLSKMENRHKKGFEACGRNLEVS 77

TR|Q8YLL5|Q8YLL5_NOSS1 KDAYSRINAIVIEGEQEAYENYIQLSQLLPDDKEDLIRLSKMESRHKKGFEACGRNLQVS 77

TR|K9TGS5|K9TGS5_9CYAN KDAYSRINAIVIEGEQEANDNYIKLAEMLPDCKDELLGLAKMELRHKKGFQACGRNLAVE 78

TR|Q3MA38|Q3MA38_ANAVT KDAYSRINAIVIEGEQEAYENYIQLSQLLPDDKEDLIRLSKMESRHKKGFEACGRNLQVS 77

TR|K9W4T2|K9W4T2_9CYAN KDAYSRINAIVIEGEQEAYENYTKLAELLPESKDELIRLSKMEMRHKKGFEACGRNLQVT 75

TR|G5J0P0|G5J0P0_CROWT KDAYSRINAIVIEGEQEAYENYIDMGELLPGDKDELIRLSKMENRHKKGFQACGKNLKVT 77

TR|Q4C7T3|Q4C7T3_CROWT KDAYSRINAIVIEGEQEAYENYIDMGELLPGDKDELIRLSKMENRHKKGFQACGKNLKVT 77

TR|I4GKL8|I4GKL8_MICAE KSAYSRINAIVIEGEYEANSNYIQLADILTDNKEELHRLAKMENRHMKGFQACGQNLKII 77

TR|T2IH71|T2IH71_CROWT KDAYSRINAIVIEGEQEAYENYIDMGELLPGDKDELIRLSKMENRHKKGFQACGKNLKVT 77

TR|T2JF13|T2JF13_CROWT KDAYSRINAIVIEGEQEAYENYIDMGELLPGDKDELIRLSKMENRHKKGFQACGKNLKVT 77

TR|K9TXF6|K9TXF6_9CYAN KDAYSRINAIVIEGEQEAYENYIRLAQMLPNLKDELIRLSKMENRHKKGFEACGRNLQVT 77

TR|K9WRL8|K9WRL8_9NOST KDAYSRINAIVIEGEQEAHDNYIKLAELLPTKQDELIRLSKMESRHKKGFEACGRNLKVT 78

TR|G6FV22|G6FV22_9CYAN KDAYSRINAIVIEGEQEAHENYLKLAELLSEHKDELIRLAKMESRHKKGFEACGRNLQVT 77

TR|K9X9I4|K9X9I4_9CHRO KDAYSRINAIVIEGEKEAHENYIRLAELLPEHKEELIRLSKMENRHKKGFEACGRNLQVT 78

TR|K9ZJT3|K9ZJT3_ANACC KDAYSRINAIVIEGEQEAHDNYIQLAELLPESKDNLIRLSKMESRHKKGFEACGRNLQVT 78

TR|B4WJ48|B4WJ48_9SYNE KDAYSRINAIVIEGELEANNNYKQLSEHLGDFKDDLLKLARMENRHMKGFQACGKNLSVN 77

TR|W5U379|W5U379_9CYAN KDAYSRVNAIVIEGEQEAHENYLKLAELLSEHKDDLIRLSKMESRHKKGFEACGRNLQVT 87

TR|K9YV86|K9YV86_DACSA KDAYSRINAIVIEGEQEAKDNYLHLIQLLPDSKDELTSLSKMEARHKKGFQACGKNLSVT 77

TR|W5U417|W5U417_9CYAN KDAYSRVNAIVIEGEQEAHENYLKLAELLSEHKDDLIRLSKMESRHKKGFEACGRNLQVT 87

TR|A0A068MTA8|A0A068MTA8_SYNY4 KDAYSRINAIVIEGEQEAYSNYLQMAELLPEDKEELVRLAKMENRHKKGFQACGNNLKVS 77

TR|Q112R8|Q112R8_TRIEI KDAYSRINAIVIEGEQEAYKNYIKLAEMLPDEKDELIKLSKMENRHKKGFEACGRNLHVT 97

TR|D7E2W1|D7E2W1_NOSA0 KDAYSRINAIVIEGEQEAHENYITLAKLLPESKEELMRLSKMESRHKKGFEACGRNLQVT 78

TR|B1WR71|B1WR71_CYAA5 KDAYSRINAIVIEGEQEAYQNYLDMAQLLPEDEAELIRLSKMENRHKKGFQACGKNLNVT 77

TR|F7US88|F7US88_SYNYG KDAYSRINAIVIEGEQEAYSNYLQMAELLPEDKEELTRLAKMENRHKKGFQACGNNLQVN 77

TR|H0PB35|H0PB35_9SYNC KDAYSRINAIVIEGEQEAYSNYLQMAELLPEDKEELTRLAKMENRHKKGFQACGNNLQVN 77

TR|H0NYN3|H0NYN3_9SYNC KDAYSRINAIVIEGEQEAYSNYLQMAELLPEDKEELTRLAKMENRHKKGFQACGNNLQVN 77

TR|L8AP37|L8AP37_BACIU KDAYSRINAIVIEGEQEAYSNYLQMAELLPEDKEELTRLAKMENRHKKGFQACGNNLQVN 77

TR|M1LIQ1|M1LIQ1_9SYNC KDAYSRINAIVIEGEQEAYSNYLQMAELLPEDKEELTRLAKMENRHKKGFQACGNNLQVN 77

TR|H0PG18|H0PG18_9SYNC KDAYSRINAIVIEGEQEAYSNYLQMAELLPEDKEELTRLAKMENRHKKGFQACGNNLQVN 77

TR|K9PTP6|K9PTP6_9CYAN KDSYSRINAIVIEGEQEAYDNYIKLAELLPENQAELIRLSKMESRHKKGFEACGRNLQVV 77

TR|X5JQD7|X5JQD7_9NOST KDAYSRINAIVIEGEQEAHENYITLAELLPEHSEQLIRLSKMENRHKKGFEACGRNLQVT 77

SP|Q55688|ALDEC_SYNY3 KDAYSRINAIVIEGEQEAYSNYLQMAELLPEDKEELTRLAKMENRHKKGFQACGNNLQVN 77

TR|K9ULL9|K9ULL9_9CHRO KDAYSRIDAIVIEGEQEAHENYITLSGLLPDFQDDLVKLSKMELRHKKGFEACARNLAVT 77

TR|K7WTY7|K7WTY7_9NOST KDAYSRINAIVIEGEQEAHENYMTLAKLLPAQEEELIRLSKMESRHKKGFEACGRNLKVT 78

TR|K9Z5G1|K9Z5G1_CYAAP KDAYSRINAIVIEGEQEAHDNYMDMIPMLPNHEKELASLAKMEFRHKKGFQSCGRNLSVT 76

TR|A3INE1|A3INE1_9CHRO KDAYSRINAIVIEGEQEAHQNYIDMAQLLPEDEAELIRLSKMENRHKKGFQACGKNLDVT 77

TR|D4TRY1|D4TRY1_9NOST KDAYSRINAIVIEGEQEAHENYLTLGELLPPNKEELSKLSKMESRHKKGFESCGRNLSVN 78

TR|V5V382|V5V382_9CHRO KDAYSRINAIVIEGEQKACDNYIDLAKLLPQHQEELTRLAKMEARHKKGFEACGRNLNVT 77

TR|B8HSZ3|B8HSZ3_CYAP4 QDAYSRIDAIVIEGEQEAHDNYLKLTELLPDCQEDLVRLAKMEARHKKGFEACGRNLKVT 77

TR|M1X0T0|M1X0T0_9NOST KDAYSRINAIVIEGEQEAHENYITLAEMLPEENEQLIRLSQMESRHKKGFEACGRNLQVA 79

SP|B2J1M1|ALDEC_NOSP7 KDAYSRINAIVIEGEQEAHENYITLAQLLPESHDELIRLSKMESRHKKGFEACGRNLAVT 78

TR|L8N0G8|L8N0G8_9CYAN RDAYSRINAIVIEGEAEAHSNYLQLAELMPEFNEELHRLAKMEDRHKKGFTACGKNLNVT 77

TR|M1WVU9|M1WVU9_9NOST KDAYSRINAIVIEGEQEAHENYITLAEMLPEENEQLIRLSQMESRHKKGFEACGRNLQVA 77

TR|B0C9L0|B0C9L0_ACAM1 KDAYSRIDGIVIEGEQEAHENYIRLGEMLPEHQDDFIRLSKMEARHKKGFEACGRNLKVT 77

TR|U3M1Y7|U3M1Y7_SYNEL --------AIVIEGEQEANDNYQKLADLLPNDKDQLIGLARMENRHKKGFEACGRNLSVT 52

TR|K9RFH5|K9RFH5_9CYAN KSAFSRINAIVVEGEQEAYENYLSLGQLLPDKKDDLVRLSKMEMRHKKGFQACAKNLEVT 77

TR|L8LLH3|L8LLH3_9CHRO KDAYSRINAIVIEGEHEAYQNYITLGDMLPDVQAELVRLAKMESRHAKGFQSCGKNLNVT 77

TR|A0A077JJ22|A0A077JJ22_9CYAN KDAYSRINAIVIKGEEEAYHNYIDIAKMLPDHKDELIRLAKMENRHKKGFQSCGKNLSVT 77

TR|B7JUH1|B7JUH1_CYAP8 KDAYSRINAIVIEGEQEAHQNYIDMAQLLPEHQEELIRLSKMENRHKKGFEACGNNLSVT 77

TR|C7QUV0|C7QUV0_CYAP0 KDAYSRINAIVIEGEQEAHQNYIDMAQLLPEHQEELIRLSKMENRHKKGFEACGNNLSVT 77

TR|U3M0X3|U3M0X3_ARTPT --------AIVIEGEQEAYDNYIKLGEMLPEEREELIRLSKMEKRHMKGFQACGRNLEVS 52

TR|D4TEY2|D4TEY2_9NOST KDAYSRINAIVIEGEQEAHENYLTLGELLPPNKEELIKLSKMESRHKKGFESCGRNLSVN 78

TR|D3EPA5|D3EPA5_ATETH KDAYSRINAIVIEGEQEAYQNYLDMVHMLPKNKDELVRLSKMENRHKTGFQACGKNLNVI 77

TR|A0A086CFQ6|A0A086CFQ6_ATETH KDAYSRINAIVIEGEQEAYQNYLDMVHMLPENKDELVRLSKMENRHKTGFQACGKNLNVV 77

.**::** * ** : : : *::** * ** :*..** :

SP|Q54764|ALDEC_SYNE7 PDMGFAQKFFERLHENFKAAAAEG--KVVTCLLIQSLIIECFAIAAYNIYIPVADAFARK 135

TR|Q5N627|Q5N627_SYNP6 PDMGFAQKFFERLHENFKAAAAEG--KVVTCLLIQSLIIECFAIAAYNIYIPVADAFARK 158

TR|Q8KPT4|Q8KPT4_SYNE7 PDMGFAQKFFERLHENFKAAAAEG--KVVTCLLIQSLIIECFAIAAYNIYIPVADAFARK 158

TR|U3M3N6|U3M3N6_9CYAN PDMGFAQKFFERLHENFKAAAAEG--KVVTCLLIQSLIIECFAIAAYNIYIPVADAFARK 110

TR|K9S7C1|K9S7C1_9CYAN PDMDFAKAFFADLHRNFQEAAAQG--KVVTCLLIQSLIIECFAIAAYNIYIPVADDFARK 135

TR|D5A1Z8|D5A1Z8_ARTPN PDMDFGREFFAQLHGNFQKAAAEG--KLVTCLLIQSLIIESFAIAAYNIYIPVADPFARK 135

TR|A0A067RRZ6|A0A067RRZ6_ARTPT PDMDFGREFFAQLHGNFQKAAAEG--KLVTCLLIQSLIIESFAIAAYNIYIPVADPFARK 135

TR|U7QM55|U7QM55_9CYAN PDMGYARQFFSQLHQNFQDAAAEG--KVVTCLLIQSLIIESFAIAAYNIYIPVADPFARK 135

TR|K1WZT6|K1WZT6_ARTPT PDMDFGREFFAKLHGNFQKAAAEG--KLVTCLLIQSLIIESFAIAAYNIYIPVADPFARK 135

TR|H1WAD2|H1WAD2_9CYAN PDMDFGREFFAKLHGNFQKAAAEG--KLVTCLLIQSLIIESFAIAAYNIYIPVADPFARK 135

TR|W6SNU7|W6SNU7_9CYAN PDMDFGREFFAKLHGNFQKAAAEG--KLVTCLLIQSLIIESFAIAAYNIYIPVADPFARK 135

TR|B5W0S5|B5W0S5_ARTMA PDMDFGREFFAKLHGNFQKAAAEG--KLVTCLLIQSLIIESFAIAAYNIYIPVADPFARK 135

TR|W5U411|W5U411_9CYAN PDMKFAKEFFAKLHDNFKQAQDEG--KIVTCLLIQSLIIETFAISAYNIYIPVADDFARK 135

TR|A0A084YC72|A0A084YC72_9CYAN PDMEFAKDFFAQLHGNFQVAAAEG--KVVTCLLIQSLIIECFAIAAYNIYIPVADDFARK 135

TR|U9WCI1|U9WCI1_9CYAN PDMEFAKIFFSKLHDNFRQARDEG--KIVTCLLIQSLIIETFAISAYNIYIPVADDFARK 135

TR|K9WII2|K9WII2_9CYAN PDMEFAKQYFSQLHGNFQTAAATG--NVVTCLLIQSLIIECFAIAAYNIYIPVADDFARK 135

TR|A0A073CMN4|A0A073CMN4_PLAAG PDMEFAQEFFSSLHQNFKDAATEG--KVVTCLLIQSLIIECFAIAAYNIYIPVADDFARK 134

TR|K8GFP8|K8GFP8_9CYAN PDMAFAREFFSSLHGNFQKAAAEG--RLVTCLLIQSLIIECFAIAAYNIYIPVADAFARK 135

TR|D8FVZ2|D8FVZ2_9CYAN PDMEFAKDFFAALHSNFQEAAAAG--KVVTCLVIQALIIECFAIAAYNIYIPVADDFARK 135

TR|B4VSW8|B4VSW8_9CYAN ADMEFAKEYFSDLHQNFQTAAASG--NIVTCLLIQSLIIECFAIAAYNIYIPVADPFARK 143

TR|W5U332|W5U332_PLAAG PDMEFAQDFFSSLHQNFKDAATEG--KVVTCLLIQSLIIECFAIAAYNIYIPVADDFARK 134

TR|K9V615|K9V615_9CYAN PDMGFAREFFAGLHKNFQDAAAEG--KVVTCLLIQSLIIECFAIAAYNIYIPVADDFARK 135

TR|K9F0G4|K9F0G4_9CYAN PDMKFAKEFFAKLHDNFKQARDEG--KVVTCLLIQSLIIETFAISAYNIYIPVADDFARK 135

TR|A0YKK4|A0YKK4_LYNSP ADMDYAHQFFSQLHQNFKDAAAQG--KVVTCLLIQSLIIESFAIAAYNIYIPVADPFARK 135

TR|I4I7T6|I4I7T6_9CHRO PDMDYAREFFSSLHENFQIAYAEG--KVVTCLLIQSLIIEAFAIAAYNIYIPVADPFARK 135

TR|I4FNH9|I4FNH9_MICAE PDMDYARAFFSSLHENFQIAYAEG--KVVTCLLIQSLIIEAFAIAAYNIYIPVADPFARK 135

TR|H2DDT7|H2DDT7_NOSS6 PDLQFAKEFFAPLHDNFKAAAATG--NVVTCLLIQSLIIECFAIAAYNIYIPVADDFARK 135

TR|G9HTG5|G9HTG5_9NOSO PDLQFAKEFFAPLHDNFKAAAATG--NVVTCLLIQSLIIECFAIAAYNIYIPVADDFARK 135

TR|I4GYG9|I4GYG9_MICAE PDMDYAREFFSSLHENFQIAYAEG--KVVTCLLIQSLIIEAFAIAAYNIYIPVADPFARK 135

TR|A0Z9X5|A0Z9X5_NODSP PDMPFAQKFFSGLHENFQKAAAEG--QVVTCLLIQSLIIECFAIAAYNIYIPVADDFARK 135

TR|K9SPY2|K9SPY2_9CYAN PDMQFAQEFFAQLHGNFQTAAAEG--DVVTCLLIQSLIIECFAIAAYNIYIPVADDFARK 137

TR|B0JY93|B0JY93_MICAN PDMDYAREFFSSLHNNFQIAYAEG--KVVTCLLIQSLIIEAFAIAAYNIYIPVADPFARK 135

TR|I4H417|I4H417_MICAE PDMDYAKEFFSSLHNNFQIAYAEG--KVVTCLLIQSLIIEAFAIAAYNIYIPVADPFARK 135

TR|I4FY74|I4FY74_MICAE PDMDYAKEFFSSLHNNFQIAYAEG--KVVTCLLIQSLIIEAFAIAAYNIYIPVADPFARK 135

TR|I4HKI6|I4HKI6_MICAE PDMDYAREFFSSLHNNFQIAYAEG--KVVTCLLIQSLIIEAFAIAAYNIYIPVADPFARK 135

TR|F5UGX9|F5UGX9_9CYAN PDMAFAKQFFSDLHRNFQTAAAEG--QIVTCLLIQSLIIECFAIAAYNIYIPVADDFARK 135

TR|L7E3D3|L7E3D3_MICAE PDMDYAREFFSSLHDNFQIAYAEG--KVVTCLLIQSLIIEAFAIAAYNIYIPVADPFARK 135

TR|K9VE50|K9VE50_9CYAN PDMAFAKEFFSELHRNFQTAAAQG--QIVTCLLIQSLIIECFAIAAYNIYIPVADDFARK 135

TR|I4IM50|I4IM50_MICAE PDMDYAREFFSSLHDNFQIAYAEG--KVVTCLLIQSLIIEAFAIAAYNIYIPVADPFARK 135

TR|K9QA48|K9QA48_9NOSO PDLQFAKEFFAPLHDNFKAGAAEN--KVVTCLLIQSLIIECFAIAAYNCYIPFADDFARK 135

TR|I4FD05|I4FD05_MICAE PDMDYAREFFSSLHDNFQIAYAEG--KVVTCLLIQSLIIEAFAIAAYNIYIPVADPFARK 135

TR|K9Y9U4|K9Y9U4_HALP7 PDMEFAQEFFAQLHQNFQDALAEG--KIVTCLLIQALIIECFAISAYNIYIPVADPFARK 135

TR|A8YJD6|A8YJD6_MICAE PDMEYAKEFFSSLHNNFQIAYAEG--KVVTCLLIQSLIIEAFAIAAYNIYIPVADPFARK 135

TR|L8NPI8|L8NPI8_MICAE PDMEYAKEFFSSLHNNFQIAYAEG--KVVTCLLIQSLIIEAFAIAAYNIYIPVADPFARK 135

TR|S3JGS6|S3JGS6_MICAE PDMEYAKEFFSSLHNNFQIAYAEG--KIVTCLLIQSLIIEAFAIAAYNIYIPVADPFARK 135

TR|I4HLI6|I4HLI6_MICAE PDMEYAKEFFSSLHNNFQIAYAEG--KIVTCLLIQSLIIEAFAIAAYNIYIPVADPFARK 135

TR|K9QXA2|K9QXA2_NOSS7 PDMQFAKEFFAELHSNFQTAAAEG--KVVTCLLIQSLIIECFAIAAYNIYIPVADDFARK 135

TR|Q8YLL5|Q8YLL5_NOSS1 PDMEFAKEFFAGLHGNFQKAAAEG--KIVTCLLIQSLIIECFAIAAYNIYIPVADDFARK 135

TR|K9TGS5|K9TGS5_9CYAN PDMEFAREFFSGLHQNFQTAAAEG--KVVTCLLIQALIIESFAISAYNIYIPVADPFARK 136

TR|Q3MA38|Q3MA38_ANAVT PDIEFAKEFFAGLHGNFQKAAAEG--KVVTCLLIQSLIIECFAIAAYNIYIPVADDFARK 135

TR|K9W4T2|K9W4T2_9CYAN ADLEFARNFFSRLHENFKVAAEAG--EIVTCLLIQSLIIECFAIAAYNIYIPVADDFARK 133

TR|G5J0P0|G5J0P0_CROWT PDMDYAERFFSQLHGNFQTAKAEG--KIVTCLLIQSLIIEAFAIAAYNIYIPVADPFARK 135

TR|Q4C7T3|Q4C7T3_CROWT PDMDYAERFFSQLHGNFQTAKAEG--KIVTCLLIQSLIIEAFAIAAYNIYIPVADPFARK 135

TR|I4GKL8|I4GKL8_MICAE PDMEYAKEFFSSLHNNFQIAYAEG--KIVTCLLIQSLIIEAFAIAAYNIYIPVADPFARK 135

TR|T2IH71|T2IH71_CROWT PDMDYAERFFSQLHGNFQTAKAEG--KIVTCLLIQSLIIEAFAIAAYNIYIPVADPFARK 135

TR|T2JF13|T2JF13_CROWT PDMDYAERFFSQLHGNFQTAKAEG--KIVTCLLIQSLIIEAFAIAAYNIYIPVADPFARK 135

TR|K9TXF6|K9TXF6_9CYAN PDMQFAREFFSGLHQNFQQAASAG--QVVTCLLIQSLIIECFAIAAYNIYIPCADDFARK 135

TR|K9WRL8|K9WRL8_9NOST PDMEFAQNFFLGLHQNFQTAAAAG--KVVTCLLIQSLIIECFAIAAYNIYIPVADEFARK 136

TR|G6FV22|G6FV22_9CYAN PDIEFAKQFFAQLHHNFQVAAQEG--KIVTCLLIQSLIIECFAIAAYNIYIPVADDFARK 135

TR|K9X9I4|K9X9I4_9CHRO PDMQFAQEFFAKLHQNFQKAAESG--NVVTCLLIQSLIIECFAIAAYNIYIPVADDFARK 136

TR|K9ZJT3|K9ZJT3_ANACC PDMKFAKEFFSGLHKNFQTAAAEG--KVVTCLLIQALIIECFAIAAYNIYIPVADDFARK 136

TR|B4WJ48|B4WJ48_9SYNE PDMPFAKEFFAQLHDNFQTALAEG--KIVTCLLIQSLIIETFAISAYNIYIPVADDFARK 135

TR|W5U379|W5U379_9CYAN PDLEFAKQFFAQLHQNFQIAAQEG--KIVTCLLIQSLIIECFAIAAYNIYIPVADDFARK 145

TR|K9YV86|K9YV86_DACSA PDMEFAQEFFAELHQNFQDALAEE--KVVTCLLIQSLIIECFAISAYNIYIPVADPFARK 135

TR|W5U417|W5U417_9CYAN PDLEFAKQFFAQLHQNFQIAAQEG--KIVTCLLIQSLIIECFAIAAYNIYIPVADDFARK 145

TR|A0A068MTA8|A0A068MTA8_SYNY4 PDMPYAQEFFSGLHGNFQKAFGEG--KIVTCLLIQALIIEAFAIAAYNIYIPVADDFARK 135

TR|Q112R8|Q112R8_TRIEI PDMEFAKKFFEPLHENFQTAAATG--NVVTCLLIQSLIIECFAIAAYNIYIPVADPFARK 155

TR|D7E2W1|D7E2W1_NOSA0 PDMQFAKEFFSGLHQNFQTAAAAG--NVVTCLLIQSLIIECFAIAAYNIYIPVADDFARK 136

TR|B1WR71|B1WR71_CYAA5 PDMDYAQQFFAELHGNFQKAKAEG--KIVTCLLIQSLIIEAFAIAAYNIYIPVADPFARK 135

TR|F7US88|F7US88_SYNYG PDMPYAQEFFAGLHGNFQHAFSEG--KVVTCLLIQALIIEAFAIAAYNIYIPVADDFARK 135

TR|H0PB35|H0PB35_9SYNC PDMPYAQEFFAGLHGNFQHAFSEG--KVVTCLLIQALIIEAFAIAAYNIYIPVADDFARK 135

TR|H0NYN3|H0NYN3_9SYNC PDMPYAQEFFAGLHGNFQHAFSEG--KVVTCLLIQALIIEAFAIAAYNIYIPVADDFARK 135

TR|L8AP37|L8AP37_BACIU PDMPYAQEFFAGLHGNFQHAFSEG--KVVTCLLIQALIIEAFAIAAYNIYIPVADDFARK 135

TR|M1LIQ1|M1LIQ1_9SYNC PDMPYAQEFFAGLHGNFQHAFSEG--KVVTCLLIQALIIEAFAIAAYNIYIPVADDFARK 135

TR|H0PG18|H0PG18_9SYNC PDMPYAQEFFAGLHGNFQHAFSEG--KVVTCLLIQALIIEAFAIAAYNIYIPVADDFARK 135

TR|K9PTP6|K9PTP6_9CYAN PDLEFAKAFFSGLHGNFQAAAAAG--KVVTCLLIQSLIIECFAIAAYNIYIPVADDFARK 135

TR|X5JQD7|X5JQD7_9NOST PDMKFAQDFFADLHQNFQTAAAEG--KIVTCLLIQSLIIECFAIAAYNIYIPVADDFARK 135

SP|Q55688|ALDEC_SYNY3 PDMPYAQEFFAGLHGNFQHAFSEG--KVVTCLLIQALIIEAFAIAAYNIYIPVADDFARK 135

TR|K9ULL9|K9ULL9_9CHRO PDLGFAKEFFAQLHENFQVAAAAG--NIVTCLLIQSLIIECFAIAAYNIYIPVADDFAKK 135

TR|K7WTY7|K7WTY7_9NOST PDLVFAKKFFLGLHDNFQTAAEEG--KVVTCLLIQSLIIECFAIAAYNIYIPVADDFARK 136

TR|K9Z5G1|K9Z5G1_CYAAP PDMDFAQKYFAELHSNFQKAKEEG--KIVTCLLIQALIIEAFAIAAYNIYIPVADPFARK 134

TR|A3INE1|A3INE1_9CHRO PDMDYAQQFFSQLHNNFQTAKAEG--KIVTCLLIQSLIIEAFAIAAYNIYIPVADPFARK 135

TR|D4TRY1|D4TRY1_9NOST PDMEFARQFFSPLHNNFQIAAQQG--KVVTCLLIQSLIIECFAIAAYNIYIPVADPFARK 136

TR|V5V382|V5V382_9CHRO PDMGFAKAFFEKLHGNFQAALAEG--KIATCLLIQALIIECFAIAAYNIYIPMADPFARK 135

TR|B8HSZ3|B8HSZ3_CYAP4 PDMEFAQQFFADLHNNFQKAAAAN--KIATCLVIQALIIECFAIAAYNIYIPVADDFARK 135

TR|M1X0T0|M1X0T0_9NOST PDMKFAKKFFADLHHNFHTASAEG--KIVTCLLIQSLIIECFAIAAYNIYIPVADNFARK 137

SP|B2J1M1|ALDEC_NOSP7 PDLQFAKEFFSGLHQNFQTAAAEG--KVVTCLLIQSLIIECFAIAAYNIYIPVADDFARK 136

TR|L8N0G8|L8N0G8_9CYAN PDMDFAEKFFSELHDNFQKAFAIG--DIVTCLLIQSLIIECFAIAAYNIYIPVADPFARK 135

TR|M1WVU9|M1WVU9_9NOST PDIKFAKNFFADLHQNFHTASAEG--KIVTCLLIQSLIIECFAIAAYNIYIPVADNFARK 135

TR|B0C9L0|B0C9L0_ACAM1 CDLDFARRFFSDLHKNFQDAAAED--KVPTCLVIQSLIIECFAIAAYNIYIPVADDFARK 135

TR|U3M1Y7|U3M1Y7_SYNEL PDMDFAREFFSELHKNFQVAAAEG--KIVTCLLIQSLIIECFAISAYNIYIPVADDFARK 110

TR|K9RFH5|K9RFH5_9CYAN ADMEFAKEFFAQLHQNFQEAAAEG--KVVTCLLIQSLIIECFAIAAYHIFIPVADDFSRK 135

TR|L8LLH3|L8LLH3_9CHRO PDMEYAKKFFAKLHGNFQQAAAEG--KIVTCFVIQSLIIEAFAIAAYHLYIPVADPFARK 135

TR|A0A077JJ22|A0A077JJ22_9CYAN PDMEYAQKFFAQLNENFQQAKAQK--KIVTCLLIQSLIIEAFAIAAYNIYIPVADPFART 135

TR|B7JUH1|B7JUH1_CYAP8 PDMQYAQEFFSSLHGNFQKAKAEG--KIVTCLLIQSLIIEAFAIAAYNIYIPVADPFARK 135

TR|C7QUV0|C7QUV0_CYAP0 PDMQYAQEFFSSLHGNFQKAKAEG--KIVTCLLIQSLIIEAFAIAAYNIYIPVADPFARK 135

TR|U3M0X3|U3M0X3_ARTPT PDMDFGREFFAQLHGNFQKAAAEG--KLVTCLLIQSLIIESFAIAAYNIYIPVADPFARK 110

TR|D4TEY2|D4TEY2_9NOST PDMEFARQFFSPLHNNFQIAAQQPKPKVVTCLLIQSLIIECFAIAAYNIYIPVADPFARK 138

TR|D3EPA5|D3EPA5_ATETH PDMQYAKEFFSQLHENFQIAKNEK--KVVTCLLIQALIIEAFAIAAYNIYIPVADPFARK 135

TR|A0A086CFQ6|A0A086CFQ6_ATETH PDMQYAKEFFSQLHENFQIAKNEK--KVVTCLLIQALIIEAFAIAAYNIYIPVADPFARK 135

*: :.. :* *. **: . : **::**:**** ***:**. :** ** *::.

SP|Q54764|ALDEC_SYNE7 ITEGVVRDEYLHRNFGEEWLKANFDASKAELEEANRQNLPLVWLMLNEVADDARELGMER 195

TR|Q5N627|Q5N627_SYNP6 ITEGVVRDEYLHRNFGEEWLKANFDASKAELEEANRQNLPLVWLMLNEVADDARELGMER 218

TR|Q8KPT4|Q8KPT4_SYNE7 ITEGVVRDEYLHRNFGEEWLKANFDASKAELEEANRQNLPLVWLMLNEVADDARELGMER 218

TR|U3M3N6|U3M3N6_9CYAN ITEGVVRDEYLHRNFGEEWLKANFDASKAELEEANRQNLPLVWLMLNEVADDARELGMER 170

TR|K9S7C1|K9S7C1_9CYAN VTEGVVKDEYSHLNFGEEWLKANFEASKAELEEANRQNLPLVWRMLNQVEQDAKTLAMEK 195

TR|D5A1Z8|D5A1Z8_ARTPN ITEGVVKDEYEHLNFGEEWLKAHFEESKAELEEANRQNLPLVWKMLNQVEKDASILGMEK 195

TR|A0A067RRZ6|A0A067RRZ6_ARTPT ITEGVVKDEYEHLNFGEEWLKAHFEESKAELEEANRQNLPLVWKMLNQVEKDASILGMEK 195

TR|U7QM55|U7QM55_9CYAN ITEGVVQDEYMHLNFGEEWLKAHFEESKAELEEANSQNLPIVWKMLNEVEKDAHILGMEK 195

TR|K1WZT6|K1WZT6_ARTPT ITEGVVKDEYEHLNFGEEWLKAHFEESKAELEEANRQNLPLVWKMLNQVEKDASILGMEK 195

TR|H1WAD2|H1WAD2_9CYAN ITEGVVKDEYEHLNFGEEWLKAHFEESKAELEEANRQNLPLVWKMLNQVEKDASILGMEK 195

TR|W6SNU7|W6SNU7_9CYAN ITEGVVKDEYEHLNFGEEWLKAHFEESKAELEEANRQNLPLVWKMLNQVEKDASILGMEK 195

TR|B5W0S5|B5W0S5_ARTMA ITEGVVKDEYEHLNFGEEWLKAHFEESKAELEEANRQNLPLVWKMLNQVEKDASILGMEK 195

TR|W5U411|W5U411_9CYAN ITEGVVKDEYMHLNFGEEWLKANFDSAKEELEEANRQNLPLVWQMLNQVADDAAILGMEK 195

TR|A0A084YC72|A0A084YC72_9CYAN ITEGVVKDEYSHLNFGEEWLKANFETSKAELEEANRQNLPIVWQMLNRVAADAEVLAMEK 195

TR|U9WCI1|U9WCI1_9CYAN ITEGVVKDEYMHLNFGEEWLKANFETAKAELEEANRQNLPLVWQMLNEVADDAAVLGMEK 195

TR|K9WII2|K9WII2_9CYAN ITEGVVKDEYMHLNFGEVWLKDHFEESKAELEQANRQNLPLVWRMLNQVEDDAHILGMEK 195

TR|A0A073CMN4|A0A073CMN4_PLAAG ITERVVKDEYMHLNFGEEWLKANFEASKTELETANRQNLPIIWRMLNQVADDAHILGMEK 194

TR|K8GFP8|K8GFP8_9CYAN ITEGVVKDEYSHLNFGEEWLKANFEQSKAELEAANRENLPLVWQMLNQVEEDARVLGMEK 195

TR|D8FVZ2|D8FVZ2_9CYAN ITEGVVKDEYNHLNFGEEWLKAHFEESKAEVDTANRQNLPIVWRMLNQVEDDARVLGMEK 195

TR|B4VSW8|B4VSW8_9CYAN ITEGVVKDEYMHLNFGEEWLKENFEASKTELEQANKQNLPLVWRMLNQVEKDAHILGMEK 203

TR|W5U332|W5U332_PLAAG ITERVVKDEYMHLNFGEEWLKANFEASKTELETANRQNLPIIWRMLNQVADDAHILGMEK 194

TR|K9V615|K9V615_9CYAN ITEGVIKEEYMHLNFGEVWLQANFETAKAELEEANRQNLPLVWQMLNQVADDAEVLAMEK 195

TR|K9F0G4|K9F0G4_9CYAN ITEGVVKDEYMHLNFGEEWLKANFDSAKKELEEANRQNLPLVWQMLNQVADDAAILGMEK 195

TR|A0YKK4|A0YKK4_LYNSP ITEGVVDDEYMHLNFGEEWLKAHFEESKAELQEANSQNLPLVWKMLNEVENDAHILGMEK 195

TR|I4I7T6|I4I7T6_9CHRO ITEGVVKDEYLHLNFGEEWLKANFETAKKELEAANRANLPIVWRMLNQVEDDARVLGMEK 195

TR|I4FNH9|I4FNH9_MICAE ITEGVVKDEYLHLNFGEEWLKANFETAKKELEAANRANLPIVWRMLNQVEDDARVLGMEK 195

TR|H2DDT7|H2DDT7_NOSS6 ITEGVVKDEYSHLNFGEVWLKANFEASKAELEEANRQNLPIVWKMLNQVAADAEVLAMEK 195

TR|G9HTG5|G9HTG5_9NOSO ITEGVVKDEYSHLNFGEVWLKANFEASKAELEEANRQNLPIVWKMLNQVAADAEVLAMEK 195

TR|I4GYG9|I4GYG9_MICAE ITESVVKDEYLHLNFGEEWLKANFETAKEELEAANRANLPIVWRMLNQVEDDARVLGMEK 195

TR|A0Z9X5|A0Z9X5_NODSP ITEGVVKDEYSHLNFGEVWLKENFAQSKAELEAANRQNLPIVWKMLNEVENDAHVLAMEK 195

TR|K9SPY2|K9SPY2_9CYAN ITEGVVKDEYLHLNFGEEWLKANFESAKAGLEKANRQNLPIVWKMLNRVEKDAKVLGMEK 197

TR|B0JY93|B0JY93_MICAN ITEGVVKDEYLHLNFGEEWLKANFETAKEELEAANRANLPIVWRMLNQVENDARVLGMEK 195

TR|I4H417|I4H417_MICAE ITEGVVKDEYLHLNFGEEWLKANFETAKDELEAANRANLPIVWRMLNQVEDDARVLAMEK 195

TR|I4FY74|I4FY74_MICAE ITEGVVKDEYLHLNFGEEWLKANFETAKEELEAANRANLPIVWKMLNQVEDDARVLAMEK 195

TR|I4HKI6|I4HKI6_MICAE ITEGVVKDEYLHLNFGEEWLKANFETAKEELEAANRANLPIVWRMLNQVENDARVLGMEK 195

TR|F5UGX9|F5UGX9_9CYAN ITEGVVKEEYSHLNFGEVWLQAHFEESKAELEAANRQNLPIIWKLLNAVADDARVLGMEK 195

TR|L7E3D3|L7E3D3_MICAE ITESVVKDEYLHLNFGEEWLKANFETAKEELEAANRANLPIVWRMLNQVEDDARVLAMEK 195

TR|K9VE50|K9VE50_9CYAN ITEGVVKEEYSHLNFGEVWLQAHFEESKAELEAANRQNLPIIWKLLNAVADDARVLGMEK 195

TR|I4IM50|I4IM50_MICAE ITESVVKDEYLHLNFGEEWLKANFETAKEELEAANRANLPIVWRMLNQVEDDARVLAMEK 195

TR|K9QA48|K9QA48_9NOSO ITEGVVKDEYSHLNFGEVWLQQNFEASKIELEEANRQNLPIVWKMLNQVADDALVLGMEK 195

TR|I4FD05|I4FD05_MICAE ITESVVKDEYLHLNFGEEWLKANFETAKEELEAANRANIPIVWRMLNQVEDDARVLAMEK 195

TR|K9Y9U4|K9Y9U4_HALP7 ITEGVVKDEYTHLNYGEEWLKANFDSAKEELEEANRQNLPIVWKMLNQVAADANVLGMEK 195

TR|A8YJD6|A8YJD6_MICAE ITESVVKDEYLHLNFGEEWLKANFETAKEELEAANRANLPIVWRMLNQVEDDARVLAMEK 195

TR|L8NPI8|L8NPI8_MICAE ITESVVKDEYLHLNFGEEWLKANFETAKEELEAANRANLPIVWRMLNQVEDDARVLAMEK 195

TR|S3JGS6|S3JGS6_MICAE ITESVVKDEYLHLNFGEEWLKANFETAKEELEAANRANLPIVWKMLNQVEDDARVLAMEK 195

TR|I4HLI6|I4HLI6_MICAE ITESVVKDEYLHLNFGEEWLKANFETAKEELEAANRANLPIVWRMLNQVEDDARVLAMEK 195

TR|K9QXA2|K9QXA2_NOSS7 ITEGVVKDEYSHLNFGEVWLKEHFAESKAELEAANRQNLPIVWRMLNQVEADAAILAMEK 195

TR|Q8YLL5|Q8YLL5_NOSS1 ITEGVVKDEYSHLNFGEVWLQKNFAQSKAELEEANRHNLPIVWKMLNQVADDAAVLAMEK 195

TR|K9TGS5|K9TGS5_9CYAN ITEGVVKDEYSHLNFGEKWLQAHFAESKTELEQANRQNLPIVWKMLNQVTDDAKVLEMEK 196

TR|Q3MA38|Q3MA38_ANAVT ITEGVVKDEYSHLNFGEVWLQKNFAQSKAELEEANRHNLPIVWKMLNQVADDAAVLAMEK 195

TR|K9W4T2|K9W4T2_9CYAN ITEGVVKDEYTHLNFGEVWLKEHFEESKAELETANRQNLPIVWQMLNQVAKDAGVLAMEK 193

TR|G5J0P0|G5J0P0_CROWT ITENVVKDEYSHLNFGEVWLKENFEASKAELEQANKENLPIVWQMLNEVEDDAEILGMEK 195

TR|Q4C7T3|Q4C7T3_CROWT ITENVVKDEYSHLNFGEVWLKENFEASKAELEQANKENLPIVWQMLNEVEDDAEILGMEK 195

TR|I4GKL8|I4GKL8_MICAE ITESVVKDEYLHLNFGEEWLKANFETAKEELEAANRANLPIVWRMLNQVEDDARVLAMEK 195

TR|T2IH71|T2IH71_CROWT ITENVVKDEYSHLNFGEVWLKENFEASKAELEQANKENLPIVWQMLNEVEDDAEILGMEK 195

TR|T2JF13|T2JF13_CROWT ITENVVKDEYSHLNFGEVWLKENFEASKAELEQANKENLPIVWQMLNEVEDDAEILGMEK 195

TR|K9TXF6|K9TXF6_9CYAN ITEGVVKDEYTHLNFGEVWLKDNFEQSKAELEAANRQNLPIVWQMLNKVEDDARVLGMEK 195

TR|K9WRL8|K9WRL8_9NOST ITEGVVKEEYSHLNFGEVWLQENFAESKAELEEANRHNLPIVWKMLNQVADDAQVLAMEK 196

TR|G6FV22|G6FV22_9CYAN ITEGVVKDEYSHLNFGEVWLKEHFEESKAELEEANRQNLPIVWQMLNQVADDAEVLAMEK 195

TR|K9X9I4|K9X9I4_9CHRO ITEGVVKDEYSHLNFGEVWLKEHFEASKAELEQANRQNLPIVWQMLNAVENDAHTLAMEK 196

TR|K9ZJT3|K9ZJT3_ANACC ITEGVVKEEYSHLNFGEVWLQENFAESKAELETANRQNLPLVWKMLNQVADDAHVLAMEK 196

TR|B4WJ48|B4WJ48_9SYNE ITEGVVKDEYMHLNFGEEWLKANFEASKAELETANRANLPLIWKMLNQVEEDAAVLGMEK 195

TR|W5U379|W5U379_9CYAN ITEGVVKDEYSHLNFGEVWLKEHFEESKAELEEANRQNLPIVWQMLNQVADDAEVLAMEK 205

TR|K9YV86|K9YV86_DACSA ITEGVVKDEYTHLNYGEEWLKAHFDEVKDELEVANRQNLPIIWKMLNQVAADAKILGMEK 195

TR|W5U417|W5U417_9CYAN ITEGVVKDEYSHLNFGEVWLKEHFEESKAELEEANRQNLPIVWQMLNQVADDAEVLAMEK 205

TR|A0A068MTA8|A0A068MTA8_SYNY4 ITEGVVKDEYTHLNYGEEWLKANFATAKEELEQANKENLPLVWKMLNQVQGDAKVLGMEK 195

TR|Q112R8|Q112R8_TRIEI ITESVVKDEYSHLNFGEVWLKEYFEDSKQELQKANRQNLPLVWKMLNQVEKDAKTLEMEK 215

TR|D7E2W1|D7E2W1_NOSA0 ITEGVVKEEYSHLNFGEVWLKEHFAESKAELDDANRQNLPIVWQMLNQVADDARVLAMEK 196

TR|B1WR71|B1WR71_CYAA5 ITEGVVKDEYTHLNFGEVWLKEHFEASKAELEDANKENLPLVWQMLNQVEKDAEVLGMEK 195

TR|F7US88|F7US88_SYNYG ITEGVVKDEYTHLNYGEEWLKANFATAKEELEQANKENLPLVWKMLNQVQGDAKVLGMEK 195

TR|H0PB35|H0PB35_9SYNC ITEGVVKDEYTHLNYGEEWLKANFATAKEELEQANKENLPLVWKMLNQVQGDAKVLGMEK 195

TR|H0NYN3|H0NYN3_9SYNC ITEGVVKDEYTHLNYGEEWLKANFATAKEELEQANKENLPLVWKMLNQVQGDAKVLGMEK 195

TR|L8AP37|L8AP37_BACIU ITEGVVKDEYTHLNYGEEWLKANFATAKEELEQANKENLPLVWKMLNQVQGDAKVLGMEK 195

TR|M1LIQ1|M1LIQ1_9SYNC ITEGVVKDEYTHLNYGEEWLKANFATAKEELEQANKENLPLVWKMLNQVQGDAKVLGMEK 195

TR|H0PG18|H0PG18_9SYNC ITEGVVKDEYTHLNYGEEWLKANFATAKEELEQANKENLPLVWKMLNQVQGDAKVLGMEK 195

TR|K9PTP6|K9PTP6_9CYAN ITEGVVKDEYSHLKFGEVWLQEHFTEAKAELEEANRQNLPIVWKMLNQVADDAKILAMEK 195

TR|X5JQD7|X5JQD7_9NOST ITESVVKDEYSHLNFGEVWLKENFEDSKAELEEANRHNLPIVWKMLNSVENDAAILAMEK 195

SP|Q55688|ALDEC_SYNY3 ITEGVVKDEYTHLNYGEEWLKANFATAKEELEQANKENLPLVWKMLNQVQGDAKVLGMEK 195

TR|K9ULL9|K9ULL9_9CHRO ITEAVVKDEYMHLNFGEVWLHDNFEASKAELQTANRQNLPLVWKMLNNVAKDAKVLGMEK 195

TR|K7WTY7|K7WTY7_9NOST ITEGVVKEEYSHLNFGEVWLQENFTESQAELEAANRQNLPIVWKMLNEVADDAKVLAMEK 196

TR|K9Z5G1|K9Z5G1_CYAAP ITEGVVKDEYLHLNFGEEWLKANFEASKAELEEANRQNLPIIWRMLNEVEKDAKTLGMPK 194

TR|A3INE1|A3INE1_9CHRO ITEGVVKDEYTHLNFGEIWLKEHFEASKAELEEANKKNLPIVWQMLNQVEKDAEVLGMEK 195

TR|D4TRY1|D4TRY1_9NOST ITEGVVKEEYTHLNFGEVWLKEKFEASKEELEVANRENLPIVWQMLNQVAEDAKVLGMEK 196

TR|V5V382|V5V382_9CHRO ITEGVVKDEYSHLNFGEIWLKENFESVKAELEEANRANLPLVWKMLNQVEADAKVLGMEK 195

TR|B8HSZ3|B8HSZ3_CYAP4 ITENVVKDEYTHLNFGEEWLKANFDSQREEVEAANRENLPIVWRMLNQVETDAHVLGMEK 195

TR|M1X0T0|M1X0T0_9NOST ITENVVKDEYTHLNFGEVWLKENFEDSKAELEEANRQNLPIVWKMLSSVEEDAAVLAMDK 197

SP|B2J1M1|ALDEC_NOSP7 ITEGVVKEEYSHLNFGEVWLKEHFAESKAELELANRQNLPIVWKMLNQVEGDAHTMAMEK 196

TR|L8N0G8|L8N0G8_9CYAN ITEGVVKDEYLHLNFGEEWLQDHFENSRATLEQANRQNLPLVWKMLNQVETDAKVLGMEK 195

TR|M1WVU9|M1WVU9_9NOST ITENVVKDEYTHLNFGEVWLKENFEDSKAELEEANRQNLPIVWKMLSSVEEDAAVLAMDK 195

TR|B0C9L0|B0C9L0_ACAM1 ITESVVKDEYQHLNYGEEWLKAHFDDVKAEIQEANRKNLPIVWRMLNEVDKDAAVLGMEK 195

TR|U3M1Y7|U3M1Y7_SYNEL ITEGVIKDEYMHLNYGEEWLKANFEASKAELEEANKANLPLVWKMLNQVADDAQVLGMER 170

TR|K9RFH5|K9RFH5_9CYAN ITEGVVKDEYTHLNFGEVWLKEHFEESKAELEEANAQNLPIVWKMLNEVETDAEVMAMPK 195

TR|L8LLH3|L8LLH3_9CHRO ITENVVKDEYTHLNFGEEWLKGNFASAKAELEEANRENLPIVWQMLNEVAEDAAILAMEK 195

TR|A0A077JJ22|A0A077JJ22_9CYAN ITEKVVKDEYIHLNFGEVWLQKNFEISKAELEEANKSNLPIVWTMLNQVKDDAEVLGMEK 195

TR|B7JUH1|B7JUH1_CYAP8 ITEGVVKDEYTHLNFGEVWLQEHFEESKAELEEANKANLPIVWEMLNQVEGDAKVLGMEK 195

TR|C7QUV0|C7QUV0_CYAP0 ITEGVVKDEYTHLNFGEVWLQEHFEESKAELEEANKANLPIVWEMLNQVEGDAKVLGMEK 195

TR|U3M0X3|U3M0X3_ARTPT ITEGVVKDEYEHLNFGEEWLKAHFEESKAELEEANRQNLPLVWKMLNQVEKDASILGMEK 170

TR|D4TEY2|D4TEY2_9NOST ITEGVVKEEYTHLNFGEVWLKEKFEASKEELEVANRENLPIVWQMLNQVAEDAKVLGMEK 198

TR|D3EPA5|D3EPA5_ATETH ITENVVKDEYKHLNFGEVWLGENFESSKIELEEANKTNLPIVWKMLNEVEQDASILGMEK 195

TR|A0A086CFQ6|A0A086CFQ6_ATETH ITENVVKDEYKHLNFGEVWLGKNFESSKLELEEANKTNLPIVWRMLNEVEQDASVLGMEK 195

:** *: :** * ::** ** * : :: ** *:*::* :*. * ** : * :

SP|Q54764|ALDEC_SYNE7 ESLVEDFMIAYGEALENIGFTTREIMRMSAYGLAAV 231

TR|Q5N627|Q5N627_SYNP6 ESLVEDFMIAYGEALENIGFTTREIMRMSAYGLAAV 254

TR|Q8KPT4|Q8KPT4_SYNE7 ESLVEDFMIAYGEALENIGFTTREIMRMSAYGLAAV 254

TR|U3M3N6|U3M3N6_9CYAN ESLVEDFMIAYGEALENIGFTTREIMRMSAYGLAAV 206

TR|K9S7C1|K9S7C1_9CYAN DALVEDFMIQYGEALDNIGFTTREIMRMSAYGLATI 231

TR|D5A1Z8|D5A1Z8_ARTPN EALIEDFMIAYGEALSNIGFTTRDIMRMSAYGLAGV 231

TR|A0A067RRZ6|A0A067RRZ6_ARTPT EALIEDFMIAYGEALSNIGFTTRDIMRMSAYGLAGV 231

TR|U7QM55|U7QM55_9CYAN DALVEDFMIAYGEALNNMGFTTREIMRMSAYGLKGA 231

TR|K1WZT6|K1WZT6_ARTPT EALIEDFMIAYGEALSNIGFTTRDIMRMSAYGLAGV 231

TR|H1WAD2|H1WAD2_9CYAN EALIEDFMIAYGEALSNIGFTTRDIMRMSAYGLAGV 231

TR|W6SNU7|W6SNU7_9CYAN EALIEDFMIAYGEALSNIGFTTRDIMRMSAYGLAGV 231

TR|B5W0S5|B5W0S5_ARTMA EALIEDFMIAYGEALSNIGFTTRDIMRMSAYGLAGV 231

TR|W5U411|W5U411_9CYAN DALVEDFMITYGEALGNIGFSSRDVMRLSAQGLATA 231

TR|A0A084YC72|A0A084YC72_9CYAN EALVEDFMIQYGEALSNIGFTTRDIMRLSAYGLAGA 231

TR|U9WCI1|U9WCI1_9CYAN EALVEDFMITYGEALSNIGFTSREVMKLSAQGLAMA 231

TR|K9WII2|K9WII2_9CYAN DALVEDFMIAYGEALSNIGFTTRDIMRMSAYGLTAA 231

TR|A0A073CMN4|A0A073CMN4_PLAAG EALVEDFMIAYGEALSNIGFSTRDIMRMSAYGLIAA 230

TR|K8GFP8|K8GFP8_9CYAN DALVEDFMIQYGEALSNIGFTTRDVMRLSAMGLSAA 231

TR|D8FVZ2|D8FVZ2_9CYAN DALVEDFMIAYGEALSNIGFTTRDIMRMSAYGLTAA 231

TR|B4VSW8|B4VSW8_9CYAN DALVEDFMIAYGEALSNIGFTTRDIMRMSAYGLTAA 239

TR|W5U332|W5U332_PLAAG EALVEDFMIAYGEALSNIGFSTRDIMRMSAYGLIAA 230

TR|K9V615|K9V615_9CYAN DALVEDFMIQYGEALSNIGFNTREIMRLSAYGLRAA 231

TR|K9F0G4|K9F0G4_9CYAN DALVEDFMITYGEALGNIGFSSRDVMRLSAQGLATA 231

TR|A0YKK4|A0YKK4_LYNSP DALVEDFMIAYGEALNNIGFTTREIMRMSAHGLTTA 231

TR|I4I7T6|I4I7T6_9CHRO EALVEDFMISYGEALSNIGFSTRDIMRMSAYGLTAV 231

TR|I4FNH9|I4FNH9_MICAE EALVEDFMISYGEALSNIGFSTRDIMRMSAYGLTAV 231

TR|H2DDT7|H2DDT7_NOSS6 EALVEDFMIQYGEALSNIGFTTRDIMRLSAYGLTAA 231

TR|G9HTG5|G9HTG5_9NOSO EALVEDFMIQYGEALSNIGFTTRDIMRLSAYGLTAA 231

TR|I4GYG9|I4GYG9_MICAE EALVEDFMISYGEALSNIGFSTRDIMRMSAYGLTAV 231

TR|A0Z9X5|A0Z9X5_NODSP EALVEDFMIQYGETLSNIGFTTRDIMKMSAYGLTAA 231

TR|K9SPY2|K9SPY2_9CYAN EALVEDFMIAYGEALGNIGFNTRDIMKMSAMGLLPA 233

TR|B0JY93|B0JY93_MICAN EALVEDFMISYGEALSNIGFSTRDIMRMSAYGLTAV 231

TR|I4H417|I4H417_MICAE EALVEDFMISYGEALSNIGFSTRDIMRMSAYGLTAV 231

TR|I4FY74|I4FY74_MICAE EALVEDFMISYGEALSNIGFSTRDIMRMSAYGLTAV 231

TR|I4HKI6|I4HKI6_MICAE EALVEDFMISYGEALSNIGFSTRDIMRMSAYGLTAV 231

TR|F5UGX9|F5UGX9_9CYAN DALIEDFMIAYGEALGNIGFNNRDIMRMSAQGLAA- 230

TR|L7E3D3|L7E3D3_MICAE EALVEDFMISYGEALSNIGFSTRDIMRMSAYGLTAV 231

TR|K9VE50|K9VE50_9CYAN DALIEDFMIAYGEALGNIGFNNRDIMRMSAQGLAA- 230

TR|I4IM50|I4IM50_MICAE EALVEDFMISYGEALSNIGFSTRDIMRMSAYGLTAV 231

TR|K9QA48|K9QA48_9NOSO DALVEDFMIQYGEALSKIGFTTRDIMRLSAYGLSAA 231

TR|I4FD05|I4FD05_MICAE EALVEDFMISYGEALSNIGFSTRDIMRMSAYGLTAV 231

TR|K9Y9U4|K9Y9U4_HALP7 DALVEDFMIAYGEALSNIGFNTREIMKMSAYGLREG 231

TR|A8YJD6|A8YJD6_MICAE EALVEDFMISYGEALNNIGFSTRDIMRMSAYGLTAV 231

TR|L8NPI8|L8NPI8_MICAE EALVEDFMISYGEALNNIGFSTRDIMRMSAYGLTAV 231

TR|S3JGS6|S3JGS6_MICAE EALVEDFMISYGEALSNIGFSTRDIMRMSAYGLTAV 231

TR|I4HLI6|I4HLI6_MICAE EALVEDFMISYGEALSNIGFSTRDIMRMSAYGLTAV 231

TR|K9QXA2|K9QXA2_NOSS7 EALVEDFMIQYGEALSNIGFTTRDIMRLSAYGLTAA 231

TR|Q8YLL5|Q8YLL5_NOSS1 EALVEDFMIQYGEALSNIGFTTRDIMRMSAYGLTAA 231

TR|K9TGS5|K9TGS5_9CYAN EALVEDFMIAYGEALSTIGFTTRDIMRMSAYGLAGA 232

TR|Q3MA38|Q3MA38_ANAVT EALVEDFMIQYGEALSNIGFTTRDIMRMSAYGLTAA 231

TR|K9W4T2|K9W4T2_9CYAN DALVEDFMIAYGEALSNIGFTTRDIMRMSAYGLTAA 229

TR|G5J0P0|G5J0P0_CROWT EALVEDFMISYGEALGNIGFSTREIMKMSAHGLAAV 231

TR|Q4C7T3|Q4C7T3_CROWT EALVEDFMISYGEALGNIGFSTREIMKMSAHGLAAV 231

TR|I4GKL8|I4GKL8_MICAE EALVEDFMISYGEALSNIGFSTRDIMRMSAYGLTAV 231

TR|T2IH71|T2IH71_CROWT EALVEDFMISYGEALGNIGFSTREIMKMSAHGLAAV 231

TR|T2JF13|T2JF13_CROWT EALVEDFMISYGEALGNIGFSTREIMKMSAHGLAAV 231

TR|K9TXF6|K9TXF6_9CYAN DALVEDFMIQYGETLANIGFSTRDIMRLSAYGLQAA 231

TR|K9WRL8|K9WRL8_9NOST EALVEDFMIQYGEALSNIGFTTRDIMRLSAYGLTAA 232

TR|G6FV22|G6FV22_9CYAN EALVEDFMIQYGEALSNIGFNTRDIMRLSAYGLRAA 231

TR|K9X9I4|K9X9I4_9CHRO EALVEDFMIQYGEALSNIGFTTRDIMRMSAYGLTAA 232

TR|K9ZJT3|K9ZJT3_ANACC EALVEDFMIQYGEALSNIGFTTRDIMRLSAYGLIPV 232

TR|B4WJ48|B4WJ48_9SYNE DALIEDFMITYGEALANIGFSARDVMRLSAQGLAAV 231

TR|W5U379|W5U379_9CYAN EALVEDFMIQYGEALSNIGFNTRDIMRLSAYGLRAA 241

TR|K9YV86|K9YV86_DACSA DALVEDFMIAYGEALSNIGFSTRDIMRMSAYGLREV 231

TR|W5U417|W5U417_9CYAN EALVEDFMIQYGEALSNIGFNTRDIMRLSAYGLRTA 241

TR|A0A068MTA8|A0A068MTA8_SYNY4 EALVEDFMISYGEALSNIGFSTREIMRMSSYGLAGV 231

TR|Q112R8|Q112R8_TRIEI EALIEDFMIAYGEALNNIGFTTGEIMRMSAYGLIAA 251

TR|D7E2W1|D7E2W1_NOSA0 EALVEDFMIQYGEALSNIGFTTRDIIRLSAYGLATV 232

TR|B1WR71|B1WR71_CYAA5 EALVEDFMISYGEALSNIGFSTREIMKMSAYGLRAA 231

TR|F7US88|F7US88_SYNYG EALVEDFMISYGEALSNIGFSTREIMRMSSYGLAGV 231

TR|H0PB35|H0PB35_9SYNC EALVEDFMISYGEALSNIGFSTREIMRMSSYGLAGV 231

TR|H0NYN3|H0NYN3_9SYNC EALVEDFMISYGEALSNIGFSTREIMRMSSYGLAGV 231

TR|L8AP37|L8AP37_BACIU EALVEDFMISYGEALSNIGFSTREIMRMSSYGLAGV 231

TR|M1LIQ1|M1LIQ1_9SYNC EALVEDFMISYGEALSNIGFSTREIMRMSSYGLAGV 231

TR|H0PG18|H0PG18_9SYNC EALVEDFMISYGEALSNIGFSTREIMRMSSYGLAGV 231

TR|K9PTP6|K9PTP6_9CYAN DALVEDFMIQYGEALSNIGFTTRDIMRLSAYGLIAA 231

TR|X5JQD7|X5JQD7_9NOST EALVEDFMIQYGEALSNIGFTTRDIMRLSAYGLRAA 231

SP|Q55688|ALDEC_SYNY3 EALVEDFMISYGEALSNIGFSTREIMRMSSYGLAGV 231

TR|K9ULL9|K9ULL9_9CHRO DALVEDFMIAYGEALSNIGFNTGEIMRMSAYGLMGA 231

TR|K7WTY7|K7WTY7_9NOST DALVEDFMIQYGEALSNIGFTTRDIMRLSAYGLATV 232

TR|K9Z5G1|K9Z5G1_CYAAP EELIEDFMISYGEALANIGFSTREIMKMSSYGLRGA 230

TR|A3INE1|A3INE1_9CHRO EALVEDFMISYGEALSNIGFSTREIMKMSSHGLSAA 231

TR|D4TRY1|D4TRY1_9NOST DALVEDFMIQYGEALSNIGFTTRDIMRLSAYGLASI 232

TR|V5V382|V5V382_9CHRO DALVEDFMIQYSGALENIGFTTREIMKMSVYGLTAA 231

TR|B8HSZ3|B8HSZ3_CYAP4 EALVESFMIQYGEALENIGFSTREIMRMSVYGLSAA 231

TR|M1X0T0|M1X0T0_9NOST EALVEDFMIQYGEALNNIGFSTRDIMRLSAYGLRKI 233

SP|B2J1M1|ALDEC_NOSP7 DALVEDFMIQYGEALSNIGFSTRDIMRLSAYGLIGA 232

TR|L8N0G8|L8N0G8_9CYAN EALVEDFMIQYGESLGKIGFNTRDIMRMSAMGLVAA 231

TR|M1WVU9|M1WVU9_9NOST EALVEDFMIQYGEALNNIGFSTRDIMRLSAYGLRKV 231

TR|B0C9L0|B0C9L0_ACAM1 EALVEDFMIQYGEALSNIGFSTGEIMRMSAYGLVAA 231

TR|U3M1Y7|U3M1Y7_SYNEL EALVEDFMIQYGEALTNIGFTTRDVMRMSAHGLAAA 206

TR|K9RFH5|K9RFH5_9CYAN DALVEDFMIQYGEALSNIGFTTRDIMRLSAYGLRAA 231

TR|L8LLH3|L8LLH3_9CHRO DALVEDFMISYGEALGNIGFNTREIMRLSSQGLKVA 231

TR|A0A077JJ22|A0A077JJ22_9CYAN EALVEDFMISYGEALSSIGFSTYDIVQMSSYGLKAT 231

TR|B7JUH1|B7JUH1_CYAP8 EALVEDFMISYGEALSNIGFSTRDIMRMSSHGLVAA 231

TR|C7QUV0|C7QUV0_CYAP0 EALVEDFMISYGEALSNIGFSTRDIMRMSSHGLVAA 231

TR|U3M0X3|U3M0X3_ARTPT EALIEDFMIAYGEALSNIGFTTRDIMRMSAYGLAGV 206

TR|D4TEY2|D4TEY2_9NOST DALVEDFMIQYGEALSNIGFTTRDIMRLSAYGLATI 234

TR|D3EPA5|D3EPA5_ATETH EALVEDFMISYGEALGNIGFSTREIMRMSSHGLRAS 231

TR|A0A086CFQ6|A0A086CFQ6_ATETH EALVEDFMISYGEALGNIGFSTREIMRMSSHGLRTS 231

: *:*.*** *. :* .:**. :::::* **
