# Supplementary material for: Identification of residues important for the activity of aldehyde-deformylating oxygenase through investigation into the structure-activity relationship
Source: BMC Biotechnol. 2017 Mar 16;17:31. doi: 10.1186/s12896-017-0351-8 (PMC5356278; doi:10.1186/s12896-017-0351-8)
Supplement: Additional file 2: — Sequence alignment of cADOs (101–150). (DOCX 20 kb) [file 12896_2017_351_MOESM2_ESM.docx]

Additional file 2 – Sequence alignment of cADOs (101-150)：

SP|Q54764|ALDEC_SYNE7 ------------------------------------------------------------

TR|U5DFA3|U5DFA3_9CHRO ------------------------------------------------------------

TR|U3M3P0|U3M3P0_9CYAN ------------------------------------------------------------

TR|K9SZC3|K9SZC3_9SYNE ------------------------------------------------------------

TR|Q8DJB4|Q8DJB4_THEEB ------------------------------------------------------------

TR|K9RTZ6|K9RTZ6_SYNP3 ------------------------------------------------------------

TR|U3M0R7|U3M0R7_9NOST ------------------------------------------------------------

TR|U3M0R6|U3M0R6_9NOST ------------------------------------------------------------

TR|U3M0X2|U3M0X2_ANAVA ------------------------------------------------------------

TR|U3M0R5|U3M0R5_9CYAN ------------------------------------------------------------

TR|U3M3N7|U3M3N7_9NOSO ------------------------------------------------------------

TR|Q2JJ13|Q2JJ13_SYNJB ------------------------------------------------------------

TR|U3M0U8|U3M0U8_9CYAN ------------------------------------------------------------

TR|Q7U5G9|Q7U5G9_SYNPX ------------------------------------------------------------

TR|A5GUD0|A5GUD0_SYNR3 ------------------------------------------------------------

TR|Q0QKF3|Q0QKF3_9SYNE ------------------------------------------------------------

TR|Q0QKK1|Q0QKK1_9SYNE ------------------------------------------------------------

TR|A4CS34|A4CS34_SYNPV ------------------------------------------------------------

TR|Q0QM11|Q0QM11_9SYNE ------------------------------------------------------------

TR|A0A076HI65|A0A076HI65_9SYNE ------------------------------------------------------------

TR|Q0QKQ4|Q0QKQ4_9SYNE ------------------------------------------------------------

TR|W0GVR9|W0GVR9_9SYNE ------------------------------------------------------------

TR|A5GJG5|A5GJG5_SYNPW ------------------------------------------------------------

TR|Q2JX62|Q2JX62_SYNJA ------------------------------------------------------------

TR|A0A076H5X1|A0A076H5X1_9SYNE ------------------------------------------------------------

TR|Q05RJ5|Q05RJ5_9SYNE ------------------------------------------------------------

TR|A0A076HNE6|A0A076HNE6_9SYNE ------------------------------------------------------------

TR|Q3ALN2|Q3ALN2_SYNSC ------------------------------------------------------------

TR|A3Z5H6|A3Z5H6_9SYNE ------------------------------------------------------------

TR|G4FIH8|G4FIH8_9SYNE ------------------------------------------------------------

TR|U5QDK0|U5QDK0_9CYAN ------------------------------------------------------------

TR|A0A081GPV5|A0A081GPV5_9CHRO ------------------------------------------------------------

TR|Q7NGM3|Q7NGM3_GLOVI ------------------------------------------------------------

TR|Q0I8M9|Q0I8M9_SYNS3 ------------------------------------------------------------

TR|K9P3X5|K9P3X5_CYAGP ------------------------------------------------------------

TR|A3YYU8|A3YYU8_9SYNE ------------------------------------------------------------

TR|K9EQR0|K9EQR0_9CYAN ------------------------------------------------------------

TR|Q3AV61|Q3AV61_SYNS9 ------------------------------------------------------------

TR|Q060S1|Q060S1_9SYNE ------------------------------------------------------------

TR|U9VZT6|U9VZT6_9CYAN ------------------------------------------------------------

TR|A2C7S0|A2C7S0_PROM3 ------------------------------------------------------------

TR|B5ILL6|B5ILL6_9CHRO ------------------------------------------------------------

SP|Q7V6D4|ALDEC_PROMM ------------------------------------------------------------

TR|A3PBQ6|A3PBQ6_PROM0 -------------------------------------------------------MHNEL 5

TR|A2BQ13|A2BQ13_PROMS -------------------------------------------------------MHNEL 5

TR|Q31C02|Q31C02_PROM9 -------------------------------------------------------MHNEL 5

TR|A9BEF6|A9BEF6_PROM4 ------------------------------------------------------------

TR|B8HLW2|B8HLW2_CYAP4 ------------------------------------------------------------

TR|Q7V2E8|Q7V2E8_PROMP ------------------------------------------------------------

TR|A2C0Z0|A2C0Z0_PROM1 ------------------------------------------------------------

TR|Q46GP5|Q46GP5_PROMT ------------------------------------------------------------

TR|Q7VD51|Q7VD51_PROMA ------------------------------------------------------------

TR|A2BVJ4|A2BVJ4_PROM5 ------------------------------------------------------------

TR|A8G3P7|A8G3P7_PROM2 ---------------------------------------------------------MNK 3

TR|A3Z6M0|A3Z6M0_9SYNE ------------------------------------------------------------

TR|A0A081GM40|A0A081GM40_9CHRO ------------------------------------------------------------

TR|K9P6M5|K9P6M5_CYAGP ------------------------------------------------------------

SP|Q54764|ALDEC_SYNE7 ---------------------------M----PQLEASLELDFQSESYKDAYSRINAIVI 29

TR|U5DFA3|U5DFA3_9CHRO ---------------------------M----QELAASTALDYDSDVYKDAYSRINAIVI 29

TR|U3M3P0|U3M3P0_9CYAN --------------------------------------------------------AIVI 4

TR|K9SZC3|K9SZC3_9SYNE ---------------------MIGQIFA----AFADENIDIDYRSDTYRDAYSRINAIVI 35

TR|Q8DJB4|Q8DJB4_THEEB ---------------------------M----TTATATPVLDYHSDRYKDAYSRINAIVI 29

TR|K9RTZ6|K9RTZ6_SYNP3 --------------------------------MSPIQAPTLDYTSETYKDAYSRINGIVI 28

TR|U3M0R7|U3M0R7_9NOST --------------------------------------------------------AIVI 4

TR|U3M0R6|U3M0R6_9NOST --------------------------------------------------------AIVI 4

TR|U3M0X2|U3M0X2_ANAVA --------------------------------------------------------AIVI 4

TR|U3M0R5|U3M0R5_9CYAN --------------------------------------------------------AIVI 4

TR|U3M3N7|U3M3N7_9NOSO --------------------------------------------------------AIVI 4

TR|Q2JJ13|Q2JJ13_SYNJB -------MNV-----------LPNTPQP----LADEGGTTLDYGSAVYRQAYSRINGIVI 38

TR|U3M0U8|U3M0U8_9CYAN --------------------------------------------------------AIVI 4

TR|Q7U5G9|Q7U5G9_SYNPX ----MTTLNAP----EAA------VVE--------GLDALPDFTTEAYKDAYSRINAIVI 38

TR|A5GUD0|A5GUD0_SYNR3 ---MASSLLDP-------------AVDG----TPVLDVELPDFTTEAYKSAYSRINAIVI 40

TR|Q0QKF3|Q0QKF3_9SYNE ----MTTLNAP----EAA------VVE--------GLDALPDFTTEAYKDAYSRINAIVI 38

TR|Q0QKK1|Q0QKK1_9SYNE ----MTTLNAP----EAA------VVE--------GLDALPDFTTEAYKDAYSRINAIVI 38

TR|A4CS34|A4CS34_SYNPV ----MPTPVTS----EVA------VLDE----QAGSASLLPDFSSEAYKDAYSRINAIVI 42

TR|Q0QM11|Q0QM11_9SYNE ----MTTLNAP----DAA------VVE--------GLDALPDFTTEAYKDAYSRINAIVI 38

TR|A0A076HI65|A0A076HI65_9SYNE ----MTTLNAP----EAS------VME--------GQDALPDFTTEAYKDAYSRINAIVI 38

TR|Q0QKQ4|Q0QKQ4_9SYNE ----MTTLNAP----EAS------VME--------GQDALPDFTTEAYKDAYSRINAIVI 38

TR|W0GVR9|W0GVR9_9SYNE ----MTTLNAP----EAP------VLE--------GQDALPDFTTEAYKDAYSRINAIVI 38

TR|A5GJG5|A5GJG5_SYNPW ----MPTPVTS----EVA------VLDG----QAGSAQALPDFSSEAYKDAYSRINAIVI 42

TR|Q2JX62|Q2JX62_SYNJA ----MAPANV-----------LPNTPPS----PTDGGGTALDYSSPRYRQAYSRINGIVI 41

TR|A0A076H5X1|A0A076H5X1_9SYNE ----MTTLNAP----TAA------VME--------GQDALPDFTTAAYKDAYSRINAIVI 38

TR|Q05RJ5|Q05RJ5_9SYNE ----MPTLNSP----EVA------AISD----QDGSASQLPDFSSAAYKDAYSRINAIVI 42

TR|A0A076HNE6|A0A076HNE6_9SYNE ----MTTLNAP----ESP------VLE--------GQDALPDFTTEAYKDAYSRINAIVI 38

TR|Q3ALN2|Q3ALN2_SYNSC ----MTTLNAP----EAP------VLE--------GQDALPDFTTAAYKDAYSRINAIVI 38

TR|A3Z5H6|A3Z5H6_9SYNE ----MPTLETS----EVA------VLED----SMASGSRLPDFTSEAYKDAYSRINAIVI 42

TR|G4FIH8|G4FIH8_9SYNE ----MSTLDST----AVA------VLDD----QQ-GLAELPDFTTDAYKDAYSRINAIVI 41

TR|U5QDK0|U5QDK0_9CYAN ----------------------MNRTDE----STAAGAPALDYRSETYRDAYSRINAIVL 34

TR|A0A081GPV5|A0A081GPV5_9CHRO ----MPTLETS----TSA---DPSLDPK----GPIEGVTLPDFSAESYKDAYSRINAIVI 45

TR|Q7NGM3|Q7NGM3_GLOVI ----------------------MN--------RTAPSSAALDYRSDTYRDAYSRINAIVL 30

TR|Q0I8M9|Q0I8M9_SYNS3 ----MPTLDST----LVA------VLDD----QQ-GLAELPDFTTDAYKDAYSRINAIVI 41

TR|K9P3X5|K9P3X5_CYAGP ----MPTVATT----TSV---DSSLDPV----GPIEGSDLPDFSTAAYKDAYSRINAIVI 45

TR|A3YYU8|A3YYU8_9SYNE ----MPSLETTIAASETASASASMAVGG----SVEQDLGLPDFSSSTYKDAYSRINAIVI 52

TR|K9EQR0|K9EQR0_9CYAN ---------------------------M----PYSAVDQTLDFHSDTYRDAFTRINGIVI 29

TR|Q3AV61|Q3AV61_SYNS9 ----MPTLNAP----EVS------VLE--------GQDALPDFTTAEYKDAYSRINAIVI 38

TR|Q060S1|Q060S1_9SYNE ------------------------MLE--------GQDALPDFTTAEYKDAYSRINAIVI 28

TR|U9VZT6|U9VZT6_9CYAN ---------------------------M----SHSVADQTLDFYSDTYRDAFTRINGIVI 29

TR|A2C7S0|A2C7S0_PROM3 ----MPTLEMP----EAA------VLDS----TVGSSEALPDFTSDAYKDAYSRINAIVI 42

TR|B5ILL6|B5ILL6_9CHRO ----MASVA----HPAVAVQPATKPADT----AAERGDGLPDFSSDTYKDAYSRINAIVI 48

SP|Q7V6D4|ALDEC_PROMM ----MPTLEMP----VAA------VLDS----TVGSSEALPDFTSDRYKDAYSRINAIVI 42

TR|A3PBQ6|A3PBQ6_PROM0 KITDMQTLESN---------KKTI-EES----TNSISLDLPDFTTDSYKDAYSRINAIVI 51

TR|A2BQ13|A2BQ13_PROMS KITDMQTLESN---------KKTI-EES----INPISLDLPDFTTDSYKDAYSRINAIVI 51

TR|Q31C02|Q31C02_PROM9 KITDMQTLETN---------TKTT-EES----IDTNSLNLPDFTTDSYKDAYSRINAIVI 51

TR|A9BEF6|A9BEF6_PROM4 ----MPTLESS---------EVA-VISD----LEGRDGSLPDFTTEQYKDAYSRINAIVI 42

TR|B8HLW2|B8HLW2_CYAP4 ---------------------------M----SDCATNPALDYYSETYRNAYRRVNGIVI 29

TR|Q7V2E8|Q7V2E8_PROMP ----MQTLESN---------KKTN---------LENSIDLPDFTTDSYKDAYSRINAIVI 38

TR|A2C0Z0|A2C0Z0_PROM1 ----MQAFASN---------NLTV--EK----EELSSDSLPDFTSESYKDAYSRINAVVI 41

TR|Q46GP5|Q46GP5_PROMT ----MQAFASN---------NLTV--EK----EELSSNSLPDFTSESYKDAYSRINAVVI 41

TR|Q7VD51|Q7VD51_PROMA ----MQTLTN---------------QVA----SADELDNLPDFSSSQYKDAYSRINAIVI 37

TR|A2BVJ4|A2BVJ4_PROM5 ----MQTLESN---------KNIQIGSS----PESDSANLPDFTTDAYKDAYSRINAIVI 43

TR|A8G3P7|A8G3P7_PROM2 SLTDMQTLESK---------KDIQLEGS----TDNDSANLPDFTTDAYKDAYSRINAIVI 50

TR|A3Z6M0|A3Z6M0_9SYNE -------------------------------------MTQLDFASAAYREAYSRINGVVI 23

TR|A0A081GM40|A0A081GM40_9CHRO -------------------------------------MANLE-LTTAYREAYSRINGLVI 22

TR|K9P6M5|K9P6M5_CYAGP -------------------------------------------MNCAYRDAYSRINALVI 17

:

SP|Q54764|ALDEC_SYNE7 EGEQEAFDNYNRLAEMLPDQRDE--LHKLAKMEQRHMKGFMA--------CGKNLSVTPD 79

TR|U5DFA3|U5DFA3_9CHRO EGEMEAKGNYTQLATLLPDSAED--LTQLAKMEGRHMKGFQA--------CGKNLSVTPD 79

TR|U3M3P0|U3M3P0_9CYAN EGEQEAHENYIKLAELLPDSQDE--LIRLSKMESRHKKGFEA--------CGRNLQVTPD 54

TR|K9SZC3|K9SZC3_9SYNE EGEQEAHDNYLQLGELLPELKTE--LAGLAKMENRHKKGFEA--------CGRNLSVIPD 85

TR|Q8DJB4|Q8DJB4_THEEB EGEQEAHDNYIDLAKLLPQHQEE--LTRLAKMEARHKKGFEA--------CGRNLSVTPD 79

TR|K9RTZ6|K9RTZ6_SYNP3 EGEKEAHENYISLTKLIPDYEED--LLRLSKMEARHKKGFEA--------CGRNLAVSPD 78

TR|U3M0R7|U3M0R7_9NOST EGEQEAYENYIRLAQMLPAHEDE--LIRLSKMESRHKKGFEA--------CGRNLQVTPD 54

TR|U3M0R6|U3M0R6_9NOST EGEQEAYENYIKLAELLPENQAD--LIRLSKMESRHKKGFEA--------CGRNLQVVPD 54

TR|U3M0X2|U3M0X2_ANAVA EGEQEAYENYIQLSQLLPDDKED--LIRLSKMESRHKKGFEA--------CGRNLQVSPD 54

TR|U3M0R5|U3M0R5_9CYAN EGEQEAHDNYIRLAQLLPDSENE--LIRLSKMESRHKKGFEA--------CGRNLQVEPD 54

TR|U3M3N7|U3M3N7_9NOSO EGEQEAHENYIQLAELLPESQTE--LIRLSKMESRHKKGFEA--------CGRNLQVTPD 54

TR|Q2JJ13|Q2JJ13_SYNJB EGEQEAHDNYLKLAEMLPEGAEE--LHKLAKMELRHMKGFQS--------CGKNLQVEPD 88

TR|U3M0U8|U3M0U8_9CYAN EGEQEAHDNYIQLSQLLPVNQEE--LIRLSKMESRHKKGFEA--------CGRNLGVVPD 54

TR|Q7U5G9|Q7U5G9_SYNPX EGEQEAHDNYISLGSLIPDQKDE--LAKLARMEMKHMKGFTS--------CGRNLGVEAD 88

TR|A5GUD0|A5GUD0_SYNR3 EGEQEAHDNYISLGTLIPDQADE--LAQLARMEMKHMKGFQA--------CGKNLSVEPD 90

TR|Q0QKF3|Q0QKF3_9SYNE EGEQEAHDNYISLGTLIPDQKDE--LAKLARMEMKHMKGFTS--------CGRNLGVEAD 88

TR|Q0QKK1|Q0QKK1_9SYNE EGEQEAHDNYISLGSLIPDQKDE--LAKLARMEMKHMKGFTS--------CGRNLGVEAD 88

TR|A4CS34|A4CS34_SYNPV EGEQEAHDNYISLGTLIPDQADE--LARLARMEMKHMKGFTS--------CGRNLGVDAD 92

TR|Q0QM11|Q0QM11_9SYNE EGEQEAHDNYIALGTLIPDQKDE--LARLARMEMKHMKGFTS--------CGRNLGVKAD 88

TR|A0A076HI65|A0A076HI65_9SYNE EGEQEAHDNYISLGTLIPDQAEE--LARLARMEMKHMKGFTS--------CGRNLGVQAD 88

TR|Q0QKQ4|Q0QKQ4_9SYNE EGEQEAHDNYISLGTLIPDQAEE--LARLARMEMKHMKGFTS--------CGRNLGVQAD 88

TR|W0GVR9|W0GVR9_9SYNE EGEQEAHDNYISLGTLIPDQAEE--LKRLARMEMKHMKGFTS--------CGRNLGVEAD 88

TR|A5GJG5|A5GJG5_SYNPW EGEQEAHDNYISLGTLIPEQADE--LARLARMEMKHMKGFMS--------CGRNLGVEAD 92

TR|Q2JX62|Q2JX62_SYNJA EGEQEAHDNYLKLAEMLPEAAEE--LRKLAKMELRHMKGFQA--------CGKNLQVEPD 91

TR|A0A076H5X1|A0A076H5X1_9SYNE EGEQEAHDNYISLGTLIPDQAEE--LTRLARMEMKHMKGFTS--------CGRNLGVEAD 88

TR|Q05RJ5|Q05RJ5_9SYNE EGEQEAHDNYISLGTLIPDQADE--LKGLARMEMKHMKGFTA--------CGNNLGVTAD 92

TR|A0A076HNE6|A0A076HNE6_9SYNE EGEQEAHDNYISLGTLIPEQAEE--LKRLARMEMKHMKGFTS--------CGRNLGVEAD 88

TR|Q3ALN2|Q3ALN2_SYNSC EGEQEAHDNYISLGTLIPEQAEE--LKRLARMEMKHMKGFTS--------CGRNLGVEAD 88

TR|A3Z5H6|A3Z5H6_9SYNE EGEQEAHDNYIALGTLIPEQKDE--LARLARMEMKHMKGFTS--------CGRNLGVEAD 92

TR|G4FIH8|G4FIH8_9SYNE EGEQEAHDNYISLGTLIPDQAEE--LAKLAKMEMKHMKGFTA--------CAKNLNVVAD 91

TR|U5QDK0|U5QDK0_9CYAN EGEREACANYLALAELLPDHADA--LKKLSAMENRHFKGFQS--------CARNLEVTPD 84

TR|A0A081GPV5|A0A081GPV5_9CHRO EGEQEAHDNYMALATLLPDQAEE--LGRLARMELKHMKGFTA--------CGNNLGVTAD 95

TR|Q7NGM3|Q7NGM3_GLOVI EGEREAHANYLTLAEMLPDHAEA--LKKLAAMENRHFKGFQS--------CARNLEVTPD 80

TR|Q0I8M9|Q0I8M9_SYNS3 EGEKEAHDNYLSLGTLIPEQAEE--LAKLAKMEMKHMKGFTA--------CAKNLDVVAD 91

TR|K9P3X5|K9P3X5_CYAGP EGEQEAHDNYMSLGTLLPDQAEE--LARLARMELKHMKGFTA--------CGNNLGVTAD 95

TR|A3YYU8|A3YYU8_9SYNE EGEQEAHDNYISLGHLIPDQAEE--LERLARMELKHKKGFTA--------CAKNLSVIAD 102

TR|K9EQR0|K9EQR0_9CYAN EGEQAAYDNFLCLADLLPEQAEE--LVRLGKMEGRHRKSFEA--------CGRNLDVIPD 79

TR|Q3AV61|Q3AV61_SYNS9 EGEQEAHDNYISLGTLIPEQADE--LSRLARMEMKHMKGFTA--------CARNLGVEAD 88

TR|Q060S1|Q060S1_9SYNE EGEQEAHDNYISLGTLIPEQAEE--LSRLARMEMKHMKGFTA--------CARNLGVEAD 78

TR|U9VZT6|U9VZT6_9CYAN EGEQAAYDNFLCLADLLPEQSEE--LTRLGKMEGRHRKSFEA--------CGRNLNVIPD 79

TR|A2C7S0|A2C7S0_PROM3 EGEQEAHDNYIAIGTLLPDHVEE--LKRLAKMEMRHKKGFTA--------CGKNLGVTAD 92

TR|B5ILL6|B5ILL6_9CHRO EGEQEAHDNYIALGTLIPDQADE--LAKLARMELKHMKGFTA--------CANNLGVTAD 98

SP|Q7V6D4|ALDEC_PROMM EGEQEAHDNYIAIGTLLPDHVEE--LKRLAKMEMRHKKGFTA--------CGKNLGVEAD 92

TR|A3PBQ6|A3PBQ6_PROM0 EGEQEAHDNYISIATLIPNELEE--LTKLARMEMKHKKGFTA--------CGRNLDVVAD 101

TR|A2BQ13|A2BQ13_PROMS EGEQEAHDNYISIATLIPNEVEE--LTKLARMEMKHKKGFTA--------CGRNLGVVAD 101

TR|Q31C02|Q31C02_PROM9 EGEQEAHDNYISIATLIPNELEE--LTKLARMELKHKKGFTA--------CGRNLGVDAD 101

TR|A9BEF6|A9BEF6_PROM4 EGEKEAHDNYVAIGTVIPEKADE--LKKLAIMELRHMKGFTA--------CGKNLGVVAD 92

TR|B8HLW2|B8HLW2_CYAP4 EGEKQAYDNFIRLAELLPEYQAE--LTRLAKMEARHQKSFVA--------CGQNLKVSPD 79

TR|Q7V2E8|Q7V2E8_PROMP EGEQEAHDNYISLATLIPNELEE--LTKLAKMELKHKRGFTA--------CGRNLGVQAD 88

TR|A2C0Z0|A2C0Z0_PROM1 EGEQEAYSNFLDLAKLIPEHADE--LVRLGKMEKKHMNGFCA--------CGRNLAVKPD 91

TR|Q46GP5|Q46GP5_PROMT EGEQEAYSNFLDLAKLIPEHADE--LVRLGKMEKKHMNGFCA--------CGRNLAVKPD 91

TR|Q7VD51|Q7VD51_PROMA EGEKEAHDNYMSIGTLIPDKADE--LKKLAVMELKHMRGFTA--------CGKNLGVKAD 87

TR|A2BVJ4|A2BVJ4_PROM5 EGEQEAYDNYISIATLLPNDSEE--LTKLAKMELKHKRGFTA--------CGKNLGVEAD 93

TR|A8G3P7|A8G3P7_PROM2 EGEQEAYDNYISIATLLPNDSEE--LTKLAKMELKHKRGFTA--------CGKNLGVEAD 100

TR|A3Z6M0|A3Z6M0_9SYNE VGEGLANRHFQMLARRIPADRDE--LQRLGRMEGDHASAFVG--------CGRNLGVVAD 73

TR|A0A081GM40|A0A081GM40_9CHRO VGEGLADRHFRLLAGLLPDERED--LEQLAAMEGRHARDFLG--------CGRSLGIRPD 72

TR|K9P6M5|K9P6M5_CYAGP VGEGLADSHFRLLAGLLPEDRDD--LMRLAAMEGHHARDFSG--------CGKQLGIRPD 67

: : . :

SP|Q54764|ALDEC_SYNE7 MGFAQKFFERLHENFKAAAAEGKVVTCLLIQSLIIECFAIAAYNIYIPVA--DAFARKIT 137

TR|U5DFA3|U5DFA3_9CHRO MDFARDYFARLHGNFQQALADGDVVACFLIQSLIIECFAIAAYNIYIPVA--DPFARKIT 137

TR|U3M3P0|U3M3P0_9CYAN LKFAKEFFSSLHQNFQTASAQGQVVTCLLIQSLIIECFAIAAYNIYIPVA--DDFARKIT 112

TR|K9SZC3|K9SZC3_9SYNE MQFAKEFFNALHGNFQKAFAITDVVTCLLIQSLIIECFAIAAYNIYIPVA--DDFARKIT 143

TR|Q8DJB4|Q8DJB4_THEEB MEFAKAFFEKLRANFQRALAEGKTATCLLIQALIIESFAIAAYNIYIPMA--DPFARKIT 137

TR|K9RTZ6|K9RTZ6_SYNP3 LDFAEKFFADLHNNFQVAAAEGKIATCLVIQALIIECFAIAAYNIYIPMA--DDFARKIT 136

TR|U3M0R7|U3M0R7_9NOST LQFAKEFFAGLHGNFQAAAAEGKVVTCLLIQSLIIECFAIAAYNIYIPVA--DDFARKIT 112

TR|U3M0R6|U3M0R6_9NOST LEFAKEFFSGLHGNFQAAAAKGKVVTCLLIQSLIIECFAIAAYNIYIPVA--DAFARKIT 112

TR|U3M0X2|U3M0X2_ANAVA IEFAKEFFAGLHGNFQKAAAEGKVVTCLLIQSLIIECFAIAAYNIYIPVA--DDFARKIT 112

TR|U3M0R5|U3M0R5_9CYAN LEFAKDFFSELHGNFQTAAAAGNVVTCLLIQSLIIECFAIAAYNIYIPVA--DDFARKIT 112

TR|U3M3N7|U3M3N7_9NOSO LQFAKEFFSGLHQNFQTAAAQGKVVTCLLIQSLIIECFAIAAYNIYIPVA--DDFARKIT 112

TR|Q2JJ13|Q2JJ13_SYNJB REFARTFFAPLRNNFQKAAAAGDLVTCLVIQSLIIECFAIAAYNIYIPVA--DEFARKIT 146

TR|U3M0U8|U3M0U8_9CYAN LQFAQEFFSGLHQNFQTAATAGKVVTCLLIQSLIIECFAIAAYNIYIPVA--DDFARKIT 112

TR|Q7U5G9|Q7U5G9_SYNPX MVFAKKFFEPLHGNFQAALKEGKVVTCLLIQALLIEAFAISAYHIYIPVA--DPFARKIT 146

TR|A5GUD0|A5GUD0_SYNR3 MVFAKEFFSDLHGNFRSALEENKVVTCLVIQALMIEAFAIAAYHIYIPVA--DPFARKIT 148

TR|Q0QKF3|Q0QKF3_9SYNE LAFAKKFFEPLHGNFQAALKEGKVVTCLLIQALLIEAFAISAYHIYIPVA--DPFARKIT 146

TR|Q0QKK1|Q0QKK1_9SYNE MVFAKTFFEPLHGNFQAALKEGKVVTCLLIQALLIEAFAISAYHIYIPVA--DPFARKIT 146

TR|A4CS34|A4CS34_SYNPV MPFAKTFFAPLHGNFQTALKDGKVVTCLLIQALLIEAFAISAYHIYIPVA--DPFARKIT 150

TR|Q0QM11|Q0QM11_9SYNE MVFAKTFFEPLHRNFQSALQEGKVVTCLLIQALLIEAFAISAYHIYIPVA--DPFARKIT 146

TR|A0A076HI65|A0A076HI65_9SYNE MAFARKFFEPLHGNFQSALKEGKVVTCLLIQALLIEAFAISAYHIYIPVA--DPFARKIT 146

TR|Q0QKQ4|Q0QKQ4_9SYNE MAFARKFFEPLHGNFQSALKEGKVVTCLLIQALLIEAFAISAYHIYIPVA--DPFARKIT 146

TR|W0GVR9|W0GVR9_9SYNE LPFAKKFFEPLHGNFQAAFKEGKVVTCLLIQALLIEAFAISAYHIYIPVA--DPFARKIT 146

TR|A5GJG5|A5GJG5_SYNPW MPFAKEFFGPLHGNFQTALKEGKVVTCLLIQALLIEAFAISAYHIYIPVA--DPFARKIT 150

TR|Q2JX62|Q2JX62_SYNJA VEFARAFFAPLRDNFQSAAAAGDLVSCFVIQSLIIECFAIAAYNIYIPVA--DDFARKIT 149

TR|A0A076H5X1|A0A076H5X1_9SYNE MAFAKTFFEPLHGNFQAAMTEGKVVTCLLIQALLIEAFAISAYHIYIPVA--DPFARKIT 146

TR|Q05RJ5|Q05RJ5_9SYNE MDFARTFFAPLHGNFQKAMKEGKVVTCLLIQALLIEAFAISAYHIYIPVA--DPFARKIT 150

TR|A0A076HNE6|A0A076HNE6_9SYNE LPFAKKFFEPLHGNFQVALKEGKVVTCLLIQALLIEAFAISAYHIYIPVA--DPFARKIT 146

TR|Q3ALN2|Q3ALN2_SYNSC LPFAKKFFEPLHGNFQAALKEGKVVTCLLIQALLIEAFAISAYHIYIPVA--DPFARKIT 146

TR|A3Z5H6|A3Z5H6_9SYNE LPFAKEFFAPLHGNFQAALQEGKVVTCLLIQALLIEAFAISAYHIYIPVA--DPFARKIT 150

TR|G4FIH8|G4FIH8_9SYNE MPFAQEFFAPLHGNFQSALKEGKVVTCLLIQALLIEAFAISAYHIYIPVA--DPFARKIT 149

TR|U5QDK0|U5QDK0_9CYAN DEFARRYFAGLDANFQRAAASGDIAACMVIQALIIECFAIAAYNIYIPVA--DPFARRVT 142

TR|A0A081GPV5|A0A081GPV5_9CHRO MPFAHEFFSPLRNNFQVALKEGKVVTCLLIQALLIEAFAISAYHIYIPVA--DPFARKIT 153

TR|Q7NGM3|Q7NGM3_GLOVI DPFARAYFEQLDGNFQQAAAEGDLTTCMVIQALIIECFAIAAYNVYIPVA--DAFARKVT 138

TR|Q0I8M9|Q0I8M9_SYNS3 MPFAQEFFAPLHGNFQSALKEGKVVTCLLIQALLIEAFAISAYHIYIPVA--DPFARKIT 149

TR|K9P3X5|K9P3X5_CYAGP MPFAQEFFSPLRNNFQAALKEGKVVTCLLIQALLIEAFAISAYHIYIPVA--DPFARKIT 153

TR|A3YYU8|A3YYU8_9SYNE MDFAKEFFSPLHGNFQAALAEGKVVTCLLIQAILIEAFAISAYHIYIPVA--DPFARKIT 160

TR|K9EQR0|K9EQR0_9CYAN LTFANQFFADLCQAFQTSATDRKVATCLLIQSLIIECFAIAAYNVYIPVA--DEFAQKVT 137

TR|Q3AV61|Q3AV61_SYNS9 MPFAKDFFGPLHGNFQVALKEGKVVTCLLIQALLIEAFAISAYHIYIPVA--DPFARKIT 146

TR|Q060S1|Q060S1_9SYNE MPFAKEFFGPLHGNFQVALKEGKVVTCLLIQALLIEAFAISAYHIYIPVA--DPFARKIT 136

TR|U9VZT6|U9VZT6_9CYAN LAFANQFFADLCQAFQTSATDRKVATCLLIQSLVIECFAISAYNVYIPVA--DEFAQKVT 137

TR|A2C7S0|A2C7S0_PROM3 MDFAREFFAPLRDNFQTALEQGKTPTCLLIQALLIEAFAISAYHTYIPVS--DPFARKIT 150

TR|B5ILL6|B5ILL6_9CHRO MPFAKEFFAPLHGNFQRALAEGKVTTCLLIQAILIEAFAISAYHIYIPVA--DPFARRIT 156

SP|Q7V6D4|ALDEC_PROMM MDFAREFFAPLRDNFQTALGQGKTPTCLLIQALLIEAFAISAYHTYIPVS--DPFARKIT 150

TR|A3PBQ6|A3PBQ6_PROM0 MEFAKKFFSKLHGNFQVALKKGNVTTCLLIQAILIEAFAISAYNVYIRVA--DPFAKKIT 159

TR|A2BQ13|A2BQ13_PROMS MDFAKKFFSKLHGNFQVALEKGNLTTCLLIQAILIEAFAISAYNVYIRVA--DPFAKKIT 159

TR|Q31C02|Q31C02_PROM9 MVFAKKFFSKLHGNFQIALEKGNLTTCLLIQAILIEAFAISAYNVYIRVA--DPFAKKIT 159

TR|A9BEF6|A9BEF6_PROM4 MEFAQRFFAPLHGNFQKALENGKITTCFLIQAILIEAFAISAYHVYIRVA--DPFAKKIT 150

TR|B8HLW2|B8HLW2_CYAP4 LDFAAQFFAELHQIFASAANAGQVATCLVVQALIIECFAIAAYNTYLPVA--DEFARKVT 137

TR|Q7V2E8|Q7V2E8_PROMP MIFAKEFFSKLHGNFQVALSNGKTTTCLLIQAILIEAFAISAYHVYIRVA--DPFAKKIT 146

TR|A2C0Z0|A2C0Z0_PROM1 MPFAKTFFSKLHNNFLEAFKVGDTTTCLLIQCILIESFAISAYHVYIRVA--DPFAKRIT 149

TR|Q46GP5|Q46GP5_PROMT MPFAKTFFSKLHNNFLEAFKVGDTTTCLLIQCILIESFAISAYHVYIRVA--DPFAKRIT 149

TR|Q7VD51|Q7VD51_PROMA IPFAEKFFSPLHGNFQKAFKEENLTTCFLIQAILIEAFAISAYHVYIRVA--DPFAKKIT 145

TR|A2BVJ4|A2BVJ4_PROM5 MSFAKEFFSKLHGNFQAALKNESLTTCLLIQAILIEAFAISAYHVYIRVA--DPFAKKIT 151

TR|A8G3P7|A8G3P7_PROM2 MPFAKEFFSKLHGNFQIALKDGNLTTCLLIQAILIEAFAISAYHVYIRVA--DPFAKKIT 158

TR|A3Z6M0|A3Z6M0_9SYNE LPLARRLFQPLHDLFKRHDHDGNRAECLVIQGLIVECFAVAAYRHYLPVA--DAYARPIT 131

TR|A0A081GM40|A0A081GM40_9CHRO LPLAHRLFAPLHRLFAEAIRAGDRVSALVIQCLIVESFAVAAYRCYQPVA--DPYAAPIL 130

TR|K9P6M5|K9P6M5_CYAGP LPLARRLFAPLHRLFREAVSSGDRVSALVIQCLIVESFAVAAYRCYQPVA--DAYAAPIL 125

SP|Q54764|ALDEC_SYNE7 EGVVRDEYLHRNFGEEWLKA------NFDASKAELEEANRQNLPLVWLMLNEVA------ 185

TR|U5DFA3|U5DFA3_9CHRO EGVVKDEYLHLNFGEVWLRE------HFEDVKERLLEANKQNLPIVWQMLNQVE------ 185

TR|U3M3P0|U3M3P0_9CYAN EGVVKDEYSHLNFGEVWLKE------HFEESKAELEEANRQNLPIVWRMLNSVA------ 160

TR|K9SZC3|K9SZC3_9SYNE EGVVKDEYLHLNFGEVWLQE------NFETAKNQLEIANRQNLPLVWKMLNQVA------ 191

TR|Q8DJB4|Q8DJB4_THEEB ESVVKDEYSHLNFGEIWLKE------HFESVKGELEEANRANLPLVWKMLNQVE------ 185

TR|K9RTZ6|K9RTZ6_SYNP3 EGVVKDEYSHLNFGEVWLQE------NFEAVKAEVETANKDNLPLVWRMLNEVE------ 184

TR|U3M0R7|U3M0R7_9NOST EGVVKDEYSHLNFGEVWLKE------HFAESKTELEEANRQNLPIVWQMLNQVA------ 160

TR|U3M0R6|U3M0R6_9NOST EGVVKDEYSHLNFGEEWLKK------HFTEAKAELEEANRQNLPIVWQMLNQVA------ 160

TR|U3M0X2|U3M0X2_ANAVA EGVVKDEYSHLNFGEVWLQK------NFAQSKAELEEANRHNLPIVWKMLNQVA------ 160

TR|U3M0R5|U3M0R5_9CYAN EGVVKDEYSHLNFGEEWLKA------NFAESKAELEAANRQNLPIVWKMLNRVA------ 160

TR|U3M3N7|U3M3N7_9NOSO EGVVKDEYSHLNFGEVWLKE------RFAESKAELEQANRQNLPLVWKMLNEVA------ 160

TR|Q2JJ13|Q2JJ13_SYNJB EGVVKDEYLHLNFGERWLGE------HFGEVKGQIEAANAQNLPLVWQMLQQVD------ 194

TR|U3M0U8|U3M0U8_9CYAN EGVVKEEYSHLNFGEVWLQA------NFAESKMELEEANRQNLPIVWTMLNQVA------ 160

TR|Q7U5G9|Q7U5G9_SYNPX EGVVKDEYTHLNYGQEWLKA------NFEASKDELFEANKANLPLIRSMLEEVA------ 194

TR|A5GUD0|A5GUD0_SYNR3 EGVVKDEYTHLNYGQEWLKA------NFDSSRDEIIEANKANLPIIRRMLEEVA------ 196

TR|Q0QKF3|Q0QKF3_9SYNE EGVVKDEYTHLNYGQEWLKA------NFEASKDELFEANKANLPLIRSMLEDVA------ 194

TR|Q0QKK1|Q0QKK1_9SYNE EGVVKDEYTHLNYGQEWLKA------NFEASKDELFEANKANLPLIRSMLEEVA------ 194

TR|A4CS34|A4CS34_SYNPV EGVVKDEYTHLNYGQEWLKA------NFDASREELMEANKVNLPLIRSMLEQVA------ 198

TR|Q0QM11|Q0QM11_9SYNE EGVVKDEYTHLNYGQEWLKA------NFEASKDELFEANKANLPLIRSMLDDVA------ 194

TR|A0A076HI65|A0A076HI65_9SYNE EGVVKDEYTHLNYGQEWLKA------NFEASKEELFEANKANLPLIRSMLEDVA------ 194

TR|Q0QKQ4|Q0QKQ4_9SYNE EGVVKDEYTHLNYGQEWLKA------NFEASKEELFEANKANLPLIRSMLEDVA------ 194

TR|W0GVR9|W0GVR9_9SYNE EGVVKDEYTHLNYGQEWLKA------NFEASKDELFEANKANLPLIRSMLEDVA------ 194

TR|A5GJG5|A5GJG5_SYNPW EGVVKDEYTHLNYGQEWLKA------NFEASREELMEANKVNLPLIRSMLEQVA------ 198

TR|Q2JX62|Q2JX62_SYNJA EGVVKDEYLHLNFGERWLGE------HFAEVKAQIEAANAQNLPLVRQMLQQVE------ 197

TR|A0A076H5X1|A0A076H5X1_9SYNE EGVVKDEYTHLNYGQEWLKA------NFEASKDELFEANKANLPLIRSMLEEVA------ 194

TR|Q05RJ5|Q05RJ5_9SYNE EGVVKDEYTHLNYGQEWLKA------NFEASKDELMEANKVNLPLIRSMLEEVA------ 198

TR|A0A076HNE6|A0A076HNE6_9SYNE EGVVKDEYTHLNYGQEWLKA------NFEASKDELFEANKANLPLIRSMLEDVA------ 194

TR|Q3ALN2|Q3ALN2_SYNSC EGVVKDEYTHLNYGQEWLKA------NFEASKNELFEANKANLPLIRSMLEDVA------ 194

TR|A3Z5H6|A3Z5H6_9SYNE EGVVKDEYTHLNYGQEWLKA------NFEASKDELMEANKANLPLIRSMLEQVA------ 198

TR|G4FIH8|G4FIH8_9SYNE EGVVKDEYTHLNYGQEWLKA------NFEASRDELMEANKVNLPLIRSMLEQVA------ 197

TR|U5QDK0|U5QDK0_9CYAN EGVVKDEYTHLNFGQQWLKD------HFEAVRPGIEQANAQNLPIVWRMLGEVE------ 190

TR|A0A081GPV5|A0A081GPV5_9CHRO EGVVKDEYTHLNYGQEWLKS------HFEESRAELEQANRENLPHVRRMLDRVA------ 201

TR|Q7NGM3|Q7NGM3_GLOVI EGVVKDEYTHLNFGQQWLKE------RFVTVREGIERANAQNLPIVWRMLNAVE------ 186

TR|Q0I8M9|Q0I8M9_SYNS3 EGVVKDEYTHLNYGQEWLKA------NFEASRDELMEANKVNLPLIRSMLEQVA------ 197

TR|K9P3X5|K9P3X5_CYAGP EGVVKDEYTHLNYGQEWLKA------HLEESRAELEQANRDNLPHVRRMLDRVA------ 201

TR|A3YYU8|A3YYU8_9SYNE EGVVKDEYTHLNYGQEWLKA------NLESSRGELEQANRVNLPLVRKMLEQVA------ 208

TR|K9EQR0|K9EQR0_9CYAN KGVVADEYHHLNFGEVWLKA------HFETVKAELETANRQVLPLIWQMLNQVD------ 185

TR|Q3AV61|Q3AV61_SYNS9 EGVVKDEYTHLNYGQEWLKA------NFEASKDEMFAANKANLPLIRSMLEGVA------ 194

TR|Q060S1|Q060S1_9SYNE EGVVKDEYTHLNYGQEWLKA------NFEASKDEMFAANKANLPLIRSMLEGVA------ 184

TR|U9VZT6|U9VZT6_9CYAN KGVVADEYHHLNFGEVWLKA------HFETVKAELETANRQVLPLIWRMLNQVE------ 185

TR|A2C7S0|A2C7S0_PROM3 EGVVKDEYTHLNYGEAWLKA------NLESCREELLEANRENLPLIRRMLDQVA------ 198

TR|B5ILL6|B5ILL6_9CHRO EGVVKDEYTHLNYGQEWLKA------NLADVREELEQANRENLPLVRKMLEQVA------ 204

SP|Q7V6D4|ALDEC_PROMM EGVVKDEYTHLNYGEAWLKA------NLESCREELLEANRENLPLIRRMLDQVA------ 198

TR|A3PBQ6|A3PBQ6_PROM0 EGVVKDEYLHLNYGQQWLKE------NLSTCKDELMEANKVNLPLIKKMLDEVA------ 207

TR|A2BQ13|A2BQ13_PROMS EGVVKDEYLHLNYGQEWLKE------NLSTCKEELMEANKVNLPLIKKMLDEVA------ 207

TR|Q31C02|Q31C02_PROM9 EGVVKDEYLHLNYGQEWLKK------NLSTCKEELMEANKVNLPLIKKMLDEVA------ 207

TR|A9BEF6|A9BEF6_PROM4 EGVVKDEYLHLNYGQEWLKA------NLATCKDELIAANKENLPLINSMLDQVA------ 198

TR|B8HLW2|B8HLW2_CYAP4 ASVVQDEYSHLNFGEVWLQN------AFEQCKDEIITANRLALPLIWKMLNQVT------ 185

TR|Q7V2E8|Q7V2E8_PROMP QGVVKDEYLHLNYGQEWLKE------NLATCKDELMEANKVNLPLIKKMLDQVS------ 194

TR|A2C0Z0|A2C0Z0_PROM1 EGVVQDEYLHLNYGQEWLKA------NLETVKKDLMRANKENLPLIKSMLDEVS------ 197

TR|Q46GP5|Q46GP5_PROMT EGVVQDEYLHLNYGQEWLKA------NLETVKKDLMRANKENLPLIKSMLDEVS------ 197

TR|Q7VD51|Q7VD51_PROMA ENVVKDEYLHLNYGQQWLKA------NLDTCKEELMKANKENLPLIKSMLDQVA------ 193

TR|A2BVJ4|A2BVJ4_PROM5 QGVVNDEYLHLNYGEKWLKE------NLSTCKDELIAANKVNLPIIKKMLDQVA------ 199

TR|A8G3P7|A8G3P7_PROM2 QGVVNDEYLHLNYGEKWLKE------NLHTCKDELIAANKVNLPLIKKMLDQVA------ 206

TR|A3Z6M0|A3Z6M0_9SYNE AAVMNDESEHLDYAETWLQR------HFDQVKARVSAVVVEALPLTLAMLQSLA------ 179

TR|A0A081GM40|A0A081GM40_9CHRO REVLEDEAEHLDYGERWLAA------WFPDVAAPIAACCERALPVALAMLQDVR------ 178

TR|K9P6M5|K9P6M5_CYAGP QTVLDDEAEHLDYGERWLAV------RFPEVAAPIAACCERAVPIVLAVLNAVR------ 173

: : : :

SP|Q54764|ALDEC_SYNE7 ------------DDARELGMERES---------LVEDFMIA----YGEALENIGFTTREI 220

TR|U5DFA3|U5DFA3_9CHRO ------------DDAAVLGMGKEE---------LIEDFMVA----YSEALSNIGFNAREI 220

TR|U3M3P0|U3M3P0_9CYAN ------------EDAHTLAMEKEA---------LVEDFMIQ----YGEALSNIGFSTRDI 195

TR|K9SZC3|K9SZC3_9SYNE ------------KDAKVLGMEKDA---------LVEDFMIQ----YGESLGKIGFNTREI 226

TR|Q8DJB4|Q8DJB4_THEEB ------------ADAKVLGMEKDA---------LVEDFMIQ----YSGALENIGFTTREI 220

TR|K9RTZ6|K9RTZ6_SYNP3 ------------KDAKVLGMDKDA---------LVEDFMIQ----YSGALENIGFTTREI 219

TR|U3M0R7|U3M0R7_9NOST ------------ADAQVLAMEKEA---------LVEDFMIQ----YGEALSNIGFSTRDI 195

TR|U3M0R6|U3M0R6_9NOST ------------DDAQTLAMEKEA---------LVEDFMIQ----YGEALSNIGFSTRDI 195

TR|U3M0X2|U3M0X2_ANAVA ------------DDAAVLAMEKEA---------LVEDFMIQ----YGEALSNIGFTTRDI 195

TR|U3M0R5|U3M0R5_9CYAN ------------DDAEVLAMEKDA---------LVEDFMIQ----YGEALSNIGFSTRDI 195

TR|U3M3N7|U3M3N7_9NOSO ------------DDAKTLAMEKEA---------LIEDFMIQ----YGEALSNIGFKTFEI 195

TR|Q2JJ13|Q2JJ13_SYNJB ------------QDVEAIYMDREA---------IVEDFMIA----YGEALANIGFSTREV 229

TR|U3M0U8|U3M0U8_9CYAN ------------ADAKTLGMEKDA---------LVEDFMIS----YGEALSHIGFTTRDI 195

TR|Q7U5G9|Q7U5G9_SYNPX ------------SDAAVLHMEKED---------LIEDFLIA----YQEALGEIGFTSRDI 229

TR|A5GUD0|A5GUD0_SYNR3 ------------DDAAELKMEKES---------LIEDFLIA----YQEALMDIGFNSRDL 231

TR|Q0QKF3|Q0QKF3_9SYNE ------------SDAAVLHMEKED---------LIEDFLIA----YQEALGEIGFTSRDI 229

TR|Q0QKK1|Q0QKK1_9SYNE ------------SDAAVLHMEKED---------LIEDFLIA----YQEALGEIGFTSRDI 229

TR|A4CS34|A4CS34_SYNPV ------------EDAAVLKMEKED---------LIEDFLIA----YQEALEQIGFTSRDI 233

TR|Q0QM11|Q0QM11_9SYNE ------------GDAAVLHMEKED---------LIEDFLIA----YQEALGEIGFTSRDI 229

TR|A0A076HI65|A0A076HI65_9SYNE ------------ADAAVLHMEKED---------LIEDFLIA----YNEALSEIGFSSRDI 229

TR|Q0QKQ4|Q0QKQ4_9SYNE ------------ADAAVLHMEKED---------LIEDFLIA----YNEALSEIGFSSRDI 229

TR|W0GVR9|W0GVR9_9SYNE ------------ADAAVLHMEKED---------LIEDFLIA----YQEALGEIGFTSRDI 229

TR|A5GJG5|A5GJG5_SYNPW ------------KDAAVLKMEKED---------LIEDFLIA----YQEALEEIGFTSRDI 233

TR|Q2JX62|Q2JX62_SYNJA ------------ADVEAIYMDREA---------IVEDFMIA----YGEALASIGFNTREV 232

TR|A0A076H5X1|A0A076H5X1_9SYNE ------------ADAAVLHMEKED---------LIEDFLIA----YQEALSEIGFSSREI 229

TR|Q05RJ5|Q05RJ5_9SYNE ------------KDAAVLHMEKED---------LIEDFLIA----YQEALNEIGFSSRDI 233

TR|A0A076HNE6|A0A076HNE6_9SYNE ------------ADAAVLHMEKED---------LIEDFLIA----YQEALGEIGFTSRDI 229

TR|Q3ALN2|Q3ALN2_SYNSC ------------ADAAVLHMEKED---------LIEDFLIA----YQEALGEIGFTSRDI 229

TR|A3Z5H6|A3Z5H6_9SYNE ------------ADAAVLQMEKED---------LIEDFLIA----YQEALCEIGFSSRDI 233

TR|G4FIH8|G4FIH8_9SYNE ------------ADASVLHMEKED---------LIEDFLIA----YQEALNEIGFSSRDI 232

TR|U5QDK0|U5QDK0_9CYAN ------------DDAATLQMDKEA---------IVEDFLIQ----YGEALSDIGFTTRDV 225

TR|A0A081GPV5|A0A081GPV5_9CHRO ------------ADASVLHMEKED---------LIEDFLIA----YQDALTDIGFNPREI 236

TR|Q7NGM3|Q7NGM3_GLOVI ------------ADTEVLQMDKEA---------IVEDFMIA----YGEALGDIGFSMRDV 221

TR|Q0I8M9|Q0I8M9_SYNS3 ------------ADASVLHMEKED---------LIEDFLIA----YQEALNEIGFSSRDI 232

TR|K9P3X5|K9P3X5_CYAGP ------------ADAAVLHMEKED---------LIEDFLIA----YQDALTDIGFTPREI 236

TR|A3YYU8|A3YYU8_9SYNE ------------GDAAVLHMDQED---------LMADFMTS----YQEALTDIGFTTREI 243

TR|K9EQR0|K9EQR0_9CYAN ------------ADLQTVGMDKQT---------LIESFLVH----YSEALKEIGFSSREI 220

TR|Q3AV61|Q3AV61_SYNS9 ------------ADAAVLHMEKED---------LIEDFLIA----YQEALNEIGFSSRDI 229

TR|Q060S1|Q060S1_9SYNE ------------ADAAVLHMEKED---------LIEDFLIA----YQEALNEIGFSSRDI 219

TR|U9VZT6|U9VZT6_9CYAN ------------ADLQTVGMDKQT---------LIEAFLVN----YSEALKEIGFNTREI 220

TR|A2C7S0|A2C7S0_PROM3 ------------GDAAVLQMDKED---------LIEDFLIA----YQESLTEIGFNTREI 233

TR|B5ILL6|B5ILL6_9CHRO ------------GDAAVLQMDKED---------LMADFLSS----YQEALMDIGFTGREI 239

SP|Q7V6D4|ALDEC_PROMM ------------GDAAVLQMDKED---------LIEDFLIA----YQESLTEIGFNTREI 233

TR|A3PBQ6|A3PBQ6_PROM0 ------------DDASVLAMDREE---------LMEEFMIA----YQDTLMEIGLDNREI 242

TR|A2BQ13|A2BQ13_PROMS ------------DDASVLAMDKEE---------LMEEFMIA----YQDTLMEIGLDNREI 242

TR|Q31C02|Q31C02_PROM9 ------------EDASVLAMDREE---------LMEEFMIA----YQDTLLEIGLDNREI 242

TR|A9BEF6|A9BEF6_PROM4 ------------NDAQVLYMEKEE---------LMEEFMIA----YQDSLMEIGLDAREI 233

TR|B8HLW2|B8HLW2_CYAP4 ------------GELRILGMDKAS---------LVEDFSTR----YGEALGQIGFKLSEI 220

TR|Q7V2E8|Q7V2E8_PROMP ------------EDASVLAMDREE---------LMEEFMIA----YQDTLLEIGLDNREI 229

TR|A2C0Z0|A2C0Z0_PROM1 ------------NDAEVLHMDKEE---------LMEEFMIA----YQDSLLEIGLDNREI 232

TR|Q46GP5|Q46GP5_PROMT ------------NDAEVLHMDKEE---------LMEEFMIA----YQDSLLEIGLDNREI 232

TR|Q7VD51|Q7VD51_PROMA ------------DDACSLSMDKEE---------LMEEFMIA----YQDSLLEIGLDSREI 228

TR|A2BVJ4|A2BVJ4_PROM5 ------------DDAATLAMDKEE---------LMEEFMIA----YQDALLEMGLDNREI 234

TR|A8G3P7|A8G3P7_PROM2 ------------EDAATLSMDKEE---------LMEEFMIA----YQDALLEMGLDNREI 241

TR|A3Z6M0|A3Z6M0_9SYNE ------------ADMRQIGMDPVE---------TLASFSEL----FREALESVGFEAVEA 214

TR|A0A081GM40|A0A081GM40_9CHRO ------------DDLTVIGIDPLD---------LVGEFVAC----FEETLEKVGFQPRQA 213

TR|K9P6M5|K9P6M5_CYAGP ------------ADLTAIGIDPAE---------LVGEFVGC----FQAAIEMVGFEPRQA 208

:

SP|Q54764|ALDEC_SYNE7 MRMSAYG----LAA--------V------------------------------------- 231

TR|U5DFA3|U5DFA3_9CHRO ARMSVHG----LAA--------A------------------------------------- 231

TR|U3M3P0|U3M3P0_9CYAN MRLSAYG----LAA--------V------------------------------------- 206

TR|K9SZC3|K9SZC3_9SYNE MKMSAMG----LVA--------A------------------------------------- 237

TR|Q8DJB4|Q8DJB4_THEEB MKMSVYG----LTG--------A------------------------------------- 231

TR|K9RTZ6|K9RTZ6_SYNP3 MKMSMHG----LVA--------A------------------------------------- 230

TR|U3M0R7|U3M0R7_9NOST MRLSAYG----LAT--------V------------------------------------- 206

TR|U3M0R6|U3M0R6_9NOST MRLSAYG----LKS--------A------------------------------------- 206

TR|U3M0X2|U3M0X2_ANAVA MRMSAYG----LTA--------A------------------------------------- 206

TR|U3M0R5|U3M0R5_9CYAN MRLSAYG----LKA--------A------------------------------------- 206

TR|U3M3N7|U3M3N7_9NOSO MRLSAHG----LTA--------A------------------------------------- 206

TR|Q2JJ13|Q2JJ13_SYNJB MRLSAQG----LRA--------A------------------------------------- 240

TR|U3M0U8|U3M0U8_9CYAN MRLSAYG----LTA--------A------------------------------------- 206

TR|Q7U5G9|Q7U5G9_SYNPX ARMAAAA----LAV---------------------------------------------- 239

TR|A5GUD0|A5GUD0_SYNR3 ARMSAAA----LVA---------------------------------------------- 241

TR|Q0QKF3|Q0QKF3_9SYNE ARMAAAA----LAV---------------------------------------------- 239

TR|Q0QKK1|Q0QKK1_9SYNE ARMAAAA----LAV---------------------------------------------- 239

TR|A4CS34|A4CS34_SYNPV ARMAAAA----LAV---------------------------------------------- 243

TR|Q0QM11|Q0QM11_9SYNE ARMAAAA----LAV---------------------------------------------- 239

TR|A0A076HI65|A0A076HI65_9SYNE ARMAAAA----LAL---------------------------------------------- 239

TR|Q0QKQ4|Q0QKQ4_9SYNE ARMAAAA----LAL---------------------------------------------- 239

TR|W0GVR9|W0GVR9_9SYNE ARMAAAA----LAV---------------------------------------------- 239

TR|A5GJG5|A5GJG5_SYNPW ARMAAAA----LSI---------------------------------------------- 243

TR|Q2JX62|Q2JX62_SYNJA MRLSAQG----LRA--------A------------------------------------- 243

TR|A0A076H5X1|A0A076H5X1_9SYNE ARMAAAA----LSI---------------------------------------------- 239

TR|Q05RJ5|Q05RJ5_9SYNE ARMAAAA----LAV---------------------------------------------- 243

TR|A0A076HNE6|A0A076HNE6_9SYNE ARMAAAA----LAV---------------------------------------------- 239

TR|Q3ALN2|Q3ALN2_SYNSC ARMAAAA----LAV---------------------------------------------- 239

TR|A3Z5H6|A3Z5H6_9SYNE ARMAAAA----LAV---------------------------------------------- 243

TR|G4FIH8|G4FIH8_9SYNE ARMAAAA----LTV---------------------------------------------- 242

TR|U5QDK0|U5QDK0_9CYAN MKMSARG----LAA--------APRA---------------------------------- 239

TR|A0A081GPV5|A0A081GPV5_9CHRO ARMAAAA----LLG---------------------------------------------- 246

TR|Q7NGM3|Q7NGM3_GLOVI MKMSARG----LAS--------APRQ---------------------------------- 235

TR|Q0I8M9|Q0I8M9_SYNS3 ARMAAAA----LSI---------------------------------------------- 242

TR|K9P3X5|K9P3X5_CYAGP ARMAAAA----LVG---------------------------------------------- 246

TR|A3YYU8|A3YYU8_9SYNE AKMATAA----LLG---------------------------------------------- 253

TR|K9EQR0|K9EQR0_9CYAN LRMASHG----LAS--------SKQR---------------------------------- 234

TR|Q3AV61|Q3AV61_SYNS9 AKMAAAA----LAI---------------------------------------------- 239

TR|Q060S1|Q060S1_9SYNE AKMAAAA----LAI---------------------------------------------- 229

TR|U9VZT6|U9VZT6_9CYAN LRMASHG----LTS--------SDQR---------------------------------- 234

TR|A2C7S0|A2C7S0_PROM3 TRMAAAA----LVS---------------------------------------------- 243

TR|B5ILL6|B5ILL6_9CHRO AKLAAAA----LVG---------------------------------------------- 249

SP|Q7V6D4|ALDEC_PROMM TRMAAAA----LVS---------------------------------------------- 243

TR|A3PBQ6|A3PBQ6_PROM0 ARMAMAA----IV----------------------------------------------- 251

TR|A2BQ13|A2BQ13_PROMS ARMAMAA----IV----------------------------------------------- 251

TR|Q31C02|Q31C02_PROM9 ARMAMAA----IV----------------------------------------------- 251

TR|A9BEF6|A9BEF6_PROM4 ARMALAA----IA----------------------------------------------- 242

TR|B8HLW2|B8HLW2_CYAP4 LSLSVQG----LQA--------VTP----------------------------------- 233

TR|Q7V2E8|Q7V2E8_PROMP ARMAMAA----IV----------------------------------------------- 238

TR|A2C0Z0|A2C0Z0_PROM1 ARMALAA----VI----------------------------------------------- 241

TR|Q46GP5|Q46GP5_PROMT ARMALAA----VI----------------------------------------------- 241

TR|Q7VD51|Q7VD51_PROMA ARMALAA----LV----------------------------------------------- 237

TR|A2BVJ4|A2BVJ4_PROM5 ARMAMAA----IV----------------------------------------------- 243

TR|A8G3P7|A8G3P7_PROM2 ARMAMAA----IV----------------------------------------------- 250

TR|A3Z6M0|A3Z6M0_9SYNE RRLLMRAAARMV------------------------------------------------ 226

TR|A0A081GM40|A0A081GM40_9CHRO RGLVARLAGRACHE--------AAPASIE---SC-------------------------- 236

TR|K9P6M5|K9P6M5_CYAGP RGLVARLAGQAVAV--------GV------------------------------------ 224
