# Supplementary material for: Identification of residues important for the activity of aldehyde-deformylating oxygenase through investigation into the structure-activity relationship
Source: BMC Biotechnol. 2017 Mar 16;17:31. doi: 10.1186/s12896-017-0351-8 (PMC5356278; doi:10.1186/s12896-017-0351-8)
Supplement: Additional file 3: Table S2. — Primers used for construction of site-directed mutants. (DOCX 16 kb) [file 12896_2017_351_MOESM3_ESM.docx]

**Additional file 3**

**Table S1 Primers used for construction of site-directed mutants**

|  | Mutants | Primer sequence (5’→3’) |
| --- | --- | --- |
| 1593 | Q49H | For: AGAAATGCTGCCGGATCACCGCGACGAACTGCA |
|  |  | Rev: TGCAGTTCGTCGCGGTGATCCGGCAGCATTTCT |
|  | N123H | For: TCGCTATCGCTGCATACCACATCTACATCCCGGTCG |
|  |  | Rev: CGACCGGGATGTAGATGTGGTATGCAGCGATAGCGA |
|  | F150Y | For: GAATACCTGCACCGTAACTACGGCGAAGAATGGCT |
|  |  | Rev: AGCCATTCTTCGCCGTAGTTACGGTGCAGGTATTC |
|  | L146T | For: TTCGTGACGAATACACGCACCGTAACTTCGGC |
|  |  | Rev: GCCGAAGTTACGGTGCGTGTATTCGTCACGAA |
|  | D143A | For: AAGGTGTGGTTCGTGCCGAATACCTGCACCG |
|  |  | Rev: CGGTGCAGGTATTCGGCACGAACCACACCTT |
|  | Y39F | For: CAGGAAGCATTTGATAACTTTAACCGTCTGGCAGA |
|  |  | Rev: TCTGCCAGACGGTTAAAGTTATCAAATGCTTCCTG |
|  | N149A | For: ACGAATACCTGCACCGTGCCTTCGGCGAAGAATGGCT |
|  |  | Rev: AGCCATTCTTCGCCGAAGGCACGGTGCAGGTATTCGT |
|  | Y122F | For: GCTTCGCTATCGCTGCATTCAACATCTACATCCCGGT |
|  |  | Rev: ACCGGGATGTAGATGTTGAATGCAGCGATAGCGAAGC |
|  | Q110L | For: GACCTGTCTGCTGATCCTGTCTCTGATTATCGAGTG |
|  |  | Rev: CACTCGATAATCAGAGACAGGATCAGCAGACAGGTC |
|  | R62A | For: GCGAAAATGGAACAGGCCCACATGAAAGGCTTCA |
|  |  | Rev: TGAAGCCTTTCATGTGGGCCTGTTCCATTTTCGC |
|  | W178R | For: GAACCTGCCTCTGGTAAGGCTGATGCTGAACGAAGT |
|  |  | Rev: ACTTCGTTCAGCATCAGCCTTACCAGAGGCAGGTTC |
| sll0208 | L148R | For: GGACGAATACACCCACCGCAACTACGGGGAAGAATG |
|  |  | Rev: CATTCTTCCCCGTAGTTGCGGTGGGTGTATTCGTCC |
|  | Y150F | For: ATACACCCACCTCAACTTCGGGGAAGAATGGCT |
|  |  | Rev: AGCCATTCTTCCCCGAAGTTGAGGTGGGTGTAT |
|  | D49H | For: GCGGAACTCTTGCCGGAACACAAAGAAGAGTTGACC |
|  |  | Rev: GGTCAACTCTTCTTTGTGTTCCGGCAAGAGTTCCGC |
|  | N123H | For: TTGCGATCGCCGCCTATCACATATATATCCCTGTG |
|  |  | Rev: GCCACAGGGATATATATGTGATAGGCGGCGATCG |
|  | T146L | For: GTCAAGGACGAATACCTCCACCTCAACTACG |
|  |  | Rev: CGTAGTTGAGGTGGAGGTATTCGTCCTTGAC |
