# Supplementary material for: Identification of residues important for the activity of aldehyde-deformylating oxygenase through investigation into the structure-activity relationship
Source: BMC Biotechnol. 2017 Mar 16;17:31. doi: 10.1186/s12896-017-0351-8 (PMC5356278; doi:10.1186/s12896-017-0351-8)
Supplement: Additional file 4: — SDS PAGE analysis of WT 1593, WT sll0208 and all variants. (DOCX 991 kb) [file 12896_2017_351_MOESM4_ESM.docx]

**Additional file 4**

**SDS PAGE analysis of WT 1593, WT sll0208 and all mutants**

**Figure S1 – WT 1593 and 1593 variants**

**
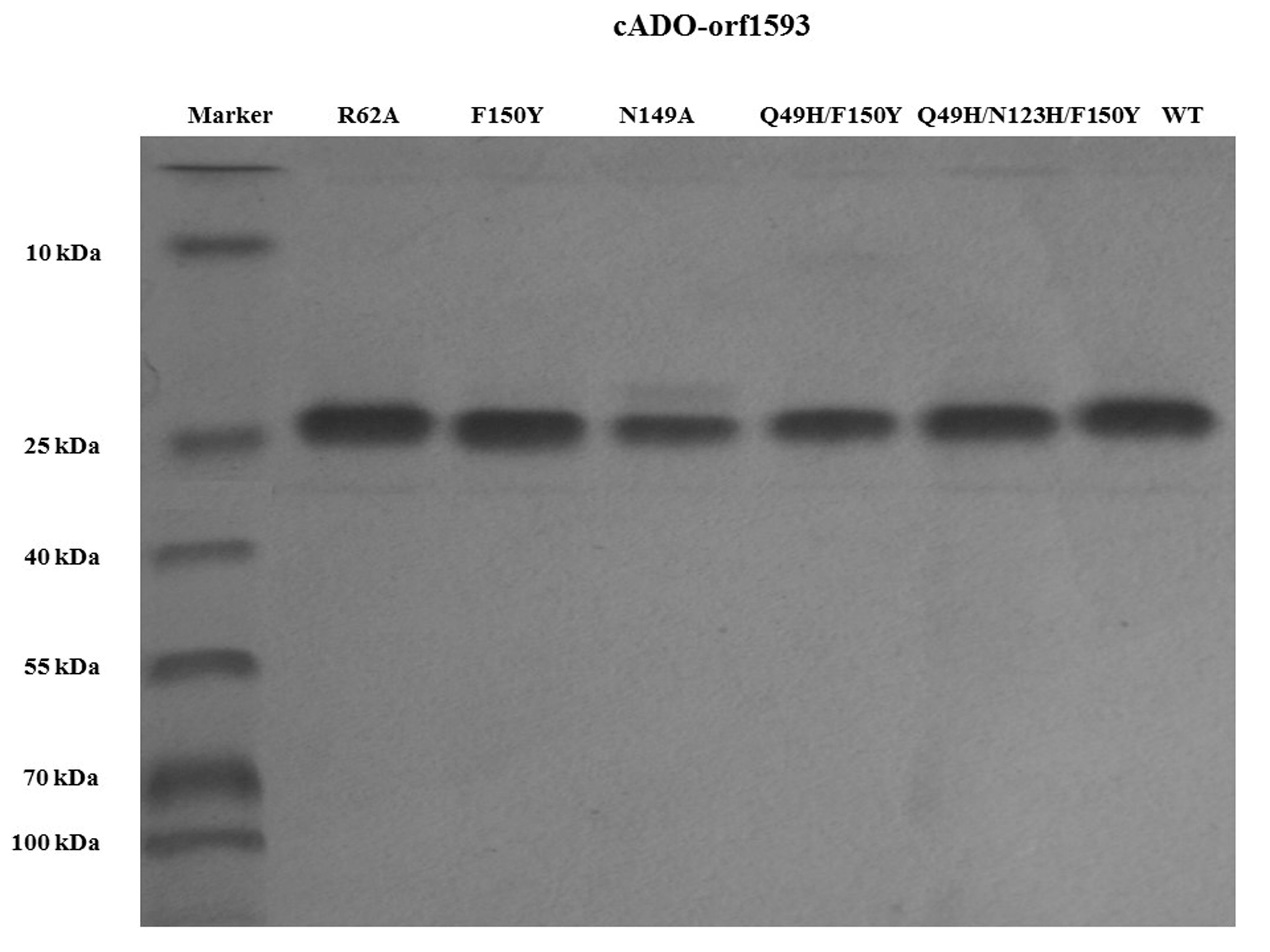
**

**Figure S2 – 1593 variants**

**
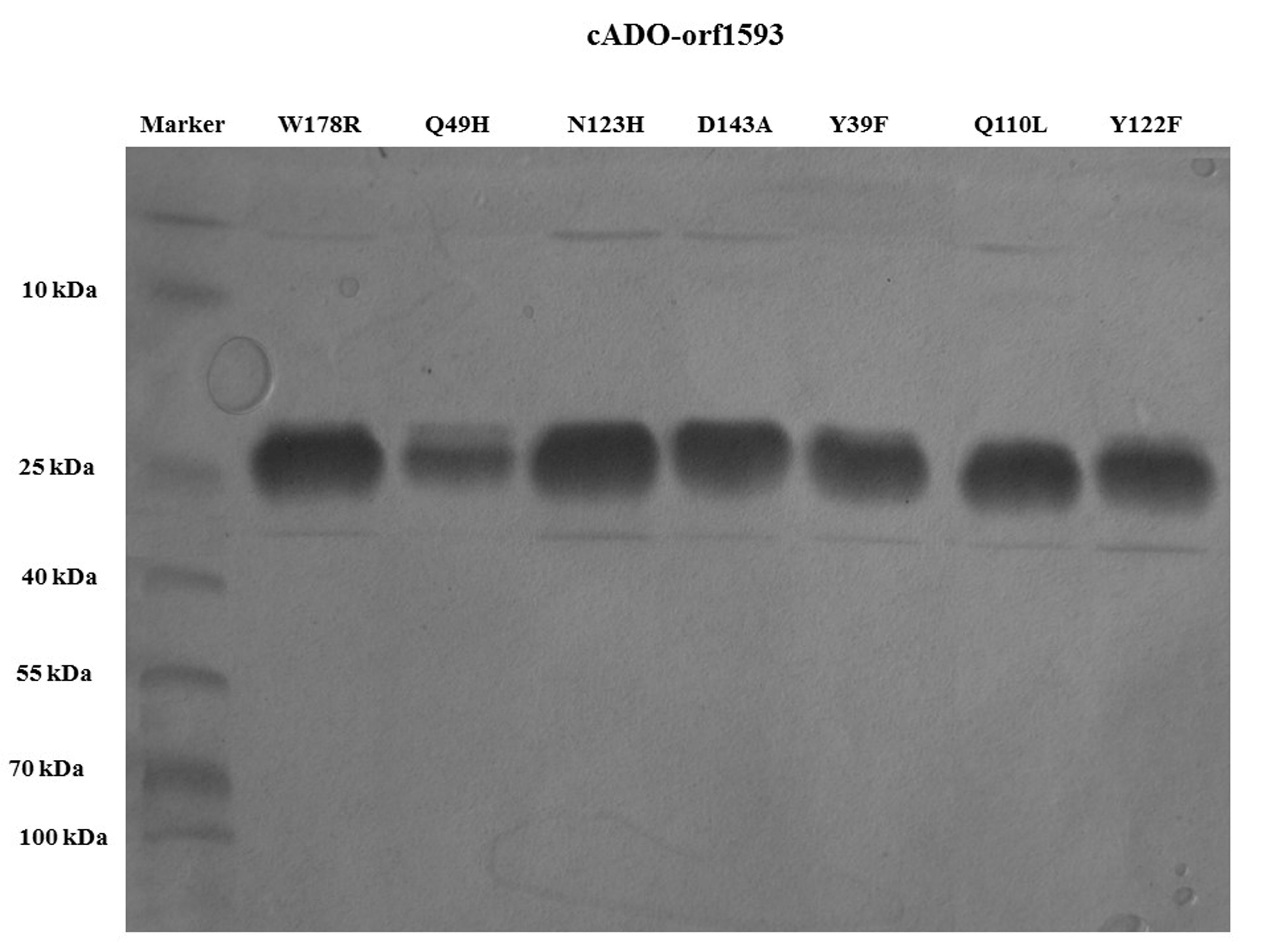
**

**Figure S3 – WT sll0208 and sll0208 variants**

**
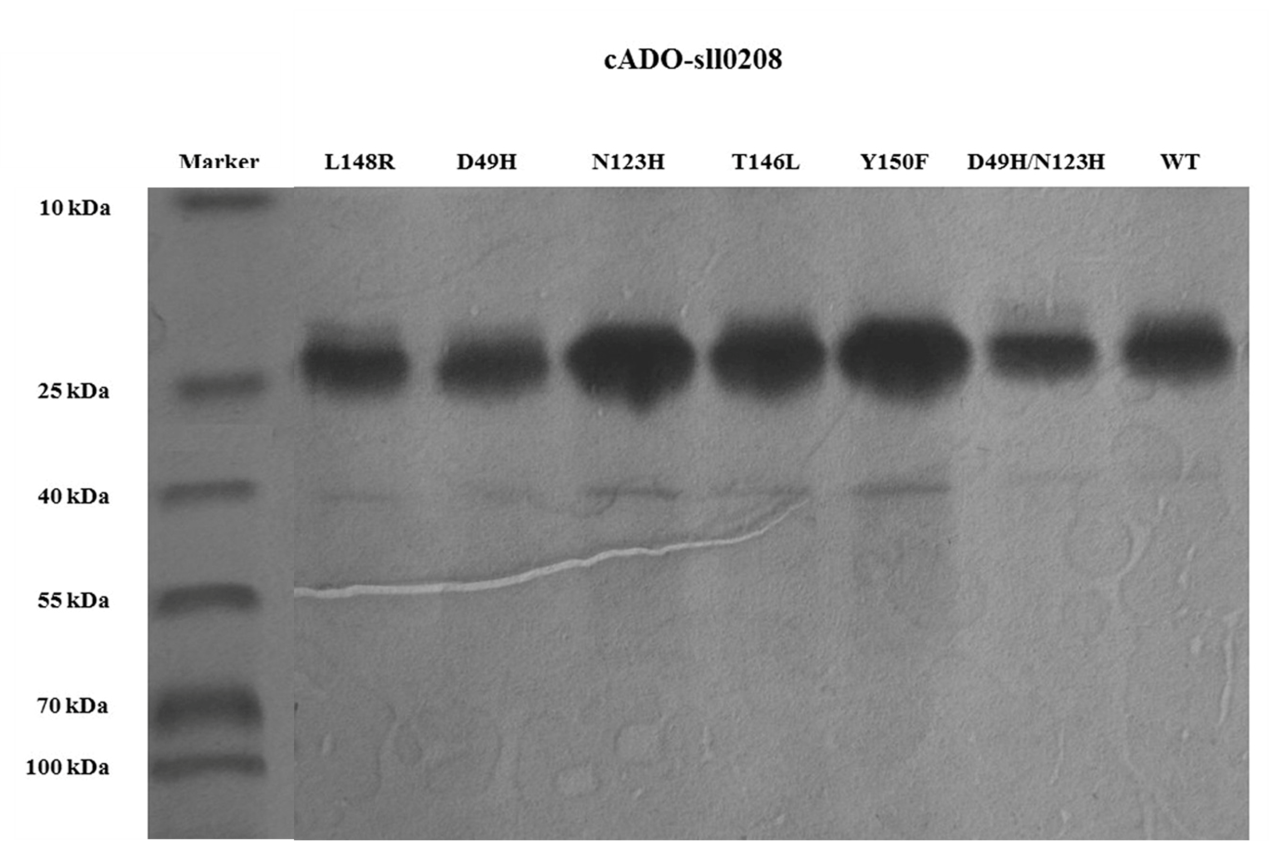
**
