# Supplementary material for: Identification of residues important for the activity of aldehyde-deformylating oxygenase through investigation into the structure-activity relationship
Source: BMC Biotechnol. 2017 Mar 16;17:31. doi: 10.1186/s12896-017-0351-8 (PMC5356278; doi:10.1186/s12896-017-0351-8)
Supplement: Additional file 5: — Original data for determination of the kinetic parameters. (DOCX 367 kb) [file 12896_2017_351_MOESM5_ESM.docx]

**Additional file 5: Original data for determination of kinetic parameters**

**Based on Michaelis-Menten equation of GraphPad Prism 5**

1. WT cADO-sll0208

1. L148R of cADO-sll0208

1. D49H/N123H of cADO-sll0208

1. L146T of cADO-1593

1. F150Y of cADO-1593

1. Q49H/F150Y of cADO-1593

1. Q49H/N123H/F150Y cADO-1593

1. W178R of cADO-1593
